# Development of cell-laden multimodular Lego-like customizable endometrial tissue assembly for successful tissue regeneration

**DOI:** 10.1186/s40824-023-00376-9

**Published:** 2023-04-21

**Authors:** Se-Ra Park, Myung Geun Kook, Soo-Rim Kim, Jin Woo Lee, Chan Hum Park, Byung-Chul Oh, YunJae Jung, In-Sun Hong

**Affiliations:** 1 Department of Health Sciences and Technology, GAIHST, Gachon University, Incheon, 21999 Republic of Korea; 2 Department of Molecular Medicine, School of Medicine, Gachon University, 7-45 Songdo-dong, Yeonsu-ku, Incheon, 406-840 Republic of Korea; 3 Department of Otolaryngology-Head and Neck Surgery, Chuncheon Sacred Heart Hospital, Hallym University College of Medicine, Chuncheon, Republic of Korea; 4 Department of Physiology, Lee Gil Ya Cancer and Diabetes Institute, Gachon University College of Medicine, Incheon, 21999 Republic of Korea; 5 Department of Microbiology, College of Medicine, Gachon University, Incheon, 21999 Korea

**Keywords:** Biomaterials, Endometrial stem cells, Lego-like tissue architecture, Multimodular tissue assembly 3D printing

## Abstract

**Background:**

The endometrium, the inner lining of the uterine cavity, plays essential roles in embryo implantation and its subsequent development. Although some positive results were preliminarily archived, the regeneration of damaged endometrial tissues by administrating stem cells only is very challenging due to the lack of specific microenvironments and their low attachment rates at the sites of injury. In this context, various biomaterial-based scaffolds have been used to overcome these limitations by providing simple structural support for cell attachment. However, these scaffold-based strategies also cannot properly reflect patient tissue-specific structural complexity and thus show only limited therapeutic effects.

**Method:**

Therefore, in the present study, we developed a customizable Lego-like multimodular endometrial tissue architecture by assembling individually fabricated tissue blocks.

**Results:**

Each tissue block was fabricated by incorporating biodegradable biomaterials and certain endometrial constituent cells. Each small tissue block was effectively fabricated by integrating conventional mold casting and 3D printing techniques. The fabricated individual tissue blocks were properly assembled into a larger customized tissue architecture. This structure not only properly mimics the patient-specific multicellular microenvironment of the endometrial tissue but also properly responds to key reproductive hormones in a manner similar to the physiological functions.

**Conclusion:**

This customizable modular tissue assembly allows easy and scalable configuration of a complex patient-specific tissue microenvironment, thus accelerating various tissue regeneration procedures.

**Supplementary Information:**

The online version contains supplementary material available at 10.1186/s40824-023-00376-9.

## Introduction

The endometrium is the mucosal internal layer of the uterus that undergoes complex regeneration and differentiation throughout the menstrual cycle [1]. Satisfactory endometrial receptivity profoundly affects successful implantation and subsequent pregnancy maintenance [2]. Severe tissue injury caused by endometrial curettage or chronic inflammation may lead to repeated pregnancy loss and infertility [3–5]. Although several fertility drug-based therapeutic approaches, such as estrogen [6] and vasodilators [7], have been used to stimulate endometrial vascularization and growth, their therapeutic efficacy is minimal. Recently, transplantations of multiple types of stem cells, such as adipose tissue stem cells [8], umbilical cord blood stem cells [9], bone marrow stem cells [10], and endometrial stem cells [11], have been proposed as ideal therapeutic alternatives to promote injured endometrial regeneration. Despite some initial promising outcomes, these stem cell-based therapies did not sufficiently regenerate damaged endometrium and the subsequent fertility rates due to their extremely low (< 1%) survival and attachment rates at the site of injury [12, 13].

To address these issues, researchers have applied various biomaterial-based scaffolds to reinforce the therapeutic efficacy of transplanted stem cells by simply supporting their physical interactions with the extracellular matrix (ECM) [14–16]. These scaffolds can provide a structural framework and regulatory substances for increasing the survival and adhesion of the transplanted cells at the site of injury to some extent [17, 18]. However, just providing a simple supportive framework and ECM components may not properly meet the complex cellular heterogeneity and physiological characteristics of the patient-specific tissue microenvironment. Therefore, to overcome the limitations of these traditional scaffold-based tissue engineering approaches, we chose the ‘Lego-like’ customizable multimodular strategy, in which each tissue component was made into an individual building block (module) and then assembled into a multimodular tissue architecture based on the patient’s specific tissue inquiries or injury conditions. Endometrial tissue consists of various cell types, such as luminal epithelial cells, stromal cells, smooth muscle cells, endothelial cells, immune cells, and resident stem cells [19]. In this context, customizable multimodular endometrial tissue assembly can be achieved through a diverse combination of five individual endometrial tissue blocks (modules) composed of each endometrial constituent cells (endometrial stem cells, myometrial cells, muscle cells, stromal cells, or vascular endothelial cells) and combined biomaterials (collagen and hyaluronic acid).

Importantly, whereas these biomaterials have high safety, flexibility, and biocompatibility, their mechanical strength is not sufficient to properly sustain the physical properties of a wide range of normal tissues. Although various chemical crosslinking agents can be used to reinforce the insufficient physical strength of biomaterials, their clinical application is limited due to the potential toxicity of residual unreacted chemical crosslinking agents [20]. For example, the widely used chemical crosslinking agents glutaraldehyde and glyoxal are known to be genotoxic and neurotoxic, respectively [21]. Therefore, in the present study, we reinforced the mechanical strength by polymerizing biomaterials with nontoxic blood coagulation factors (fibrinogen and thrombin) as natural crosslinking agents.

In addition, since each tissue block was fabricated by a mold casting process by injecting biomaterials and endometrial cells into a 3D-printed polylactic acid (PLA) mold, our Lego-like multimodular strategy requires less cost, equipment, and time than recently spotlighted 3D bioprinting techniques requiring specific infrastructure that is mostly expensive. Thus, our multimodular tissue assembly can also be mass producible, similar to plastic Lego blocks, with a low-cost. Our customizable multimodular tissue assembly consists of a variety of tissue blocks with a unified shape and standard interface for easy assembly, such as the Lego block. Moreover, the multimodular endometrial tissue assembly effectively retained various physiological characteristics of the endometrium, such as cell-specific marker expression, sex hormone responsiveness, and the secretion of reproductive hormones and growth factors. More importantly, severe degenerative tissue damage was significantly relieved by transplantation of our multimodular tissue assembly in vivo. To the best of our knowledge, this is the first report to develop a customizable Lego-like multimodular endometrial tissue assembly. The approach involves assembling different tissue blocks, each composed of specific endometrial cells and biomaterials, to reconstruct the patient’s tissue microenvironment. The researchers reinforced the mechanical strength of the biomaterials using natural crosslinking agents, avoiding the potential toxicity of traditional agents. The resulting tissue blocks retain endometrial characteristics and can effectively regenerate damaged tissue in vivo.

## Materials and methods

### 3D printing of various casting molds for the fabrications of tissue blocks and assembly sheets

As shown in Fig. 2, CAD (computer aided design) software (SolidWorks 2009, SolidWorks Corp., Concord, MA, USA) was utilized to design the casting molds for various endometrial tissue blocks and assembly sheets. After finishing designs for tissue block and assembly sheet molds, 3D CAD data were changed to 3D surface geometry data composed of triangles with their orthogonal vectors and they were transformed to the sliced data of at the Z-axis. Each sliced layer information was loaded to the digital light processing-based 3D printer (Master EV, Carima, Seoul, Korea) and the casting mold for various endometrial tissue blocks and assembly sheets were fabricated using layer-by-layer 3D printing process by the photo-polymerization mechanism of a liquid photocurable poly lactic acid (PLA).

**Fig. 1:**
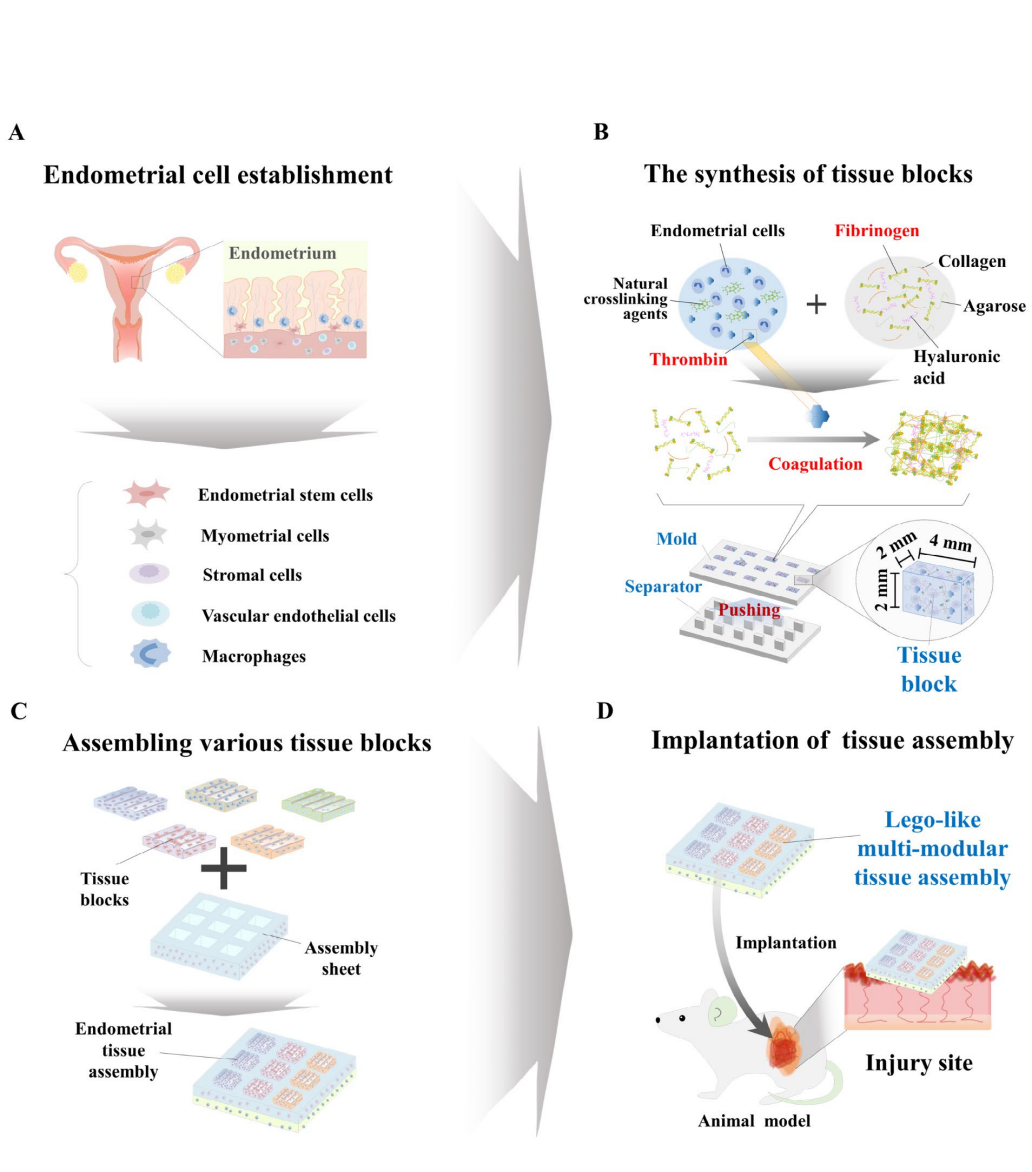
**The basic concept of a Lego-like multimodular tissue assembly recapitulates the multicellular structural complexity of human endometrial tissue.** The basic concept of this study is to develop customized multimodular endometrial tissue architectures by assembling individual tissue blocks that encapsulate certain endometrial cell types according to patient-specific tissue microenvironments or injured tissue requirements. As a first step for this object, various cell types constituting the human endometrial tissue were isolated or established **(A)**. Each tissue block (module) encapsulating individual endometrial cells was synthesized by casting using a 3D printed PLA mold to effectively and stably produce a large amount of tissue blocks. Briefly, the biomaterial mixture (collagen and hyaluronic acid) with coagulation factors (fibrinogen and thrombin) and approximately 1 × 10^6^ endometrial cells were mixed and injected into a 3D printed PLA mold and then polymerized and cooled to room temperature for 1 h. The polymerized individual tissue blocks were effectively released from the PLA mold by slowly pushing them with a separator **(B)**. Next, individually synthesized tissue blocks were assembled into the multimodular endometrium architecture by inserting them into multiple empty slots on the assembly sheet based on patient-specific tissue microenvironments or injured tissue requirements **(C)**. Finally, the multimodular endometrial tissue assembly encapsulating various endometrial cells was directly transplanted into the tissue injury mouse model, and its therapeutic effects were analyzed in vivo**(D)**

**Fig. 2:**
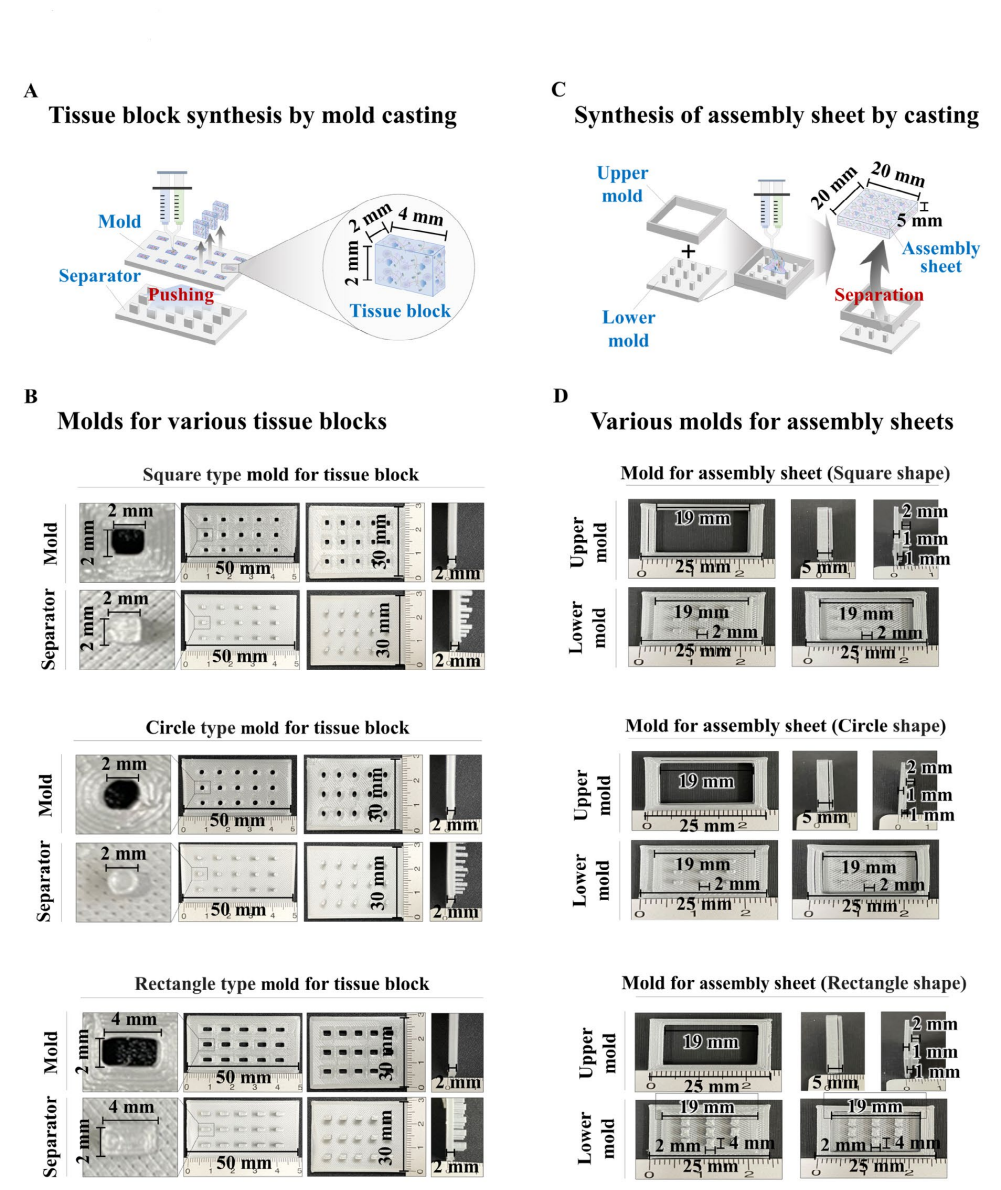
**Synthesis of a PLA-based fabricating mold for individual tissue blocks by integrating traditional casting and 3D printing techniques.** Each tissue block was synthesized by using a 3D printed fabricating mold to effectively and stably produce a large amount of tissue blocks at a much faster time and at low cost than the previous module-making methods **(A)**. Three different types of fabricating casting molds (50 × 30 × 2 mm^3^) were 3D printed using polylactic acid (PLA), and each mold can produce 15 endometrial tissue blocks at once: (1) square blocks (2 × 2 × 2 mm^3^), (2) rectangular blocks (2 × 2 × 4 mm^3^), and (3) circular blocks (2 × 2 × 2 mm^3^). We also conducted PLA-based 3D printing to develop a separator that can effectively release polymerized tissue blocks from the casting mold by pushing them **(B)**. For accurate assembly of individual tissue blocks without leaking, individual tissue blocks were properly inserted into multiple empty slots on the prefabricated assembly sheet **(C)**. The casting molds for three different types of assembly sheets (20 × 20 × 5 mm^3^), which contain exactly the same shape and size of slots as each tissue block, were 3D printed using PLA **(D)**

### Isolation and establishment of various human endometrial cell types

Human endometrial stem cells were freshly isolated from endometrial tissue biopsies of uterine fibroid patients with written informed consent document from the patients and approval of the Gachon University Institutional Review Board (IRB No: GAIRB2018-134). Endometrial tissues biopsies were mechanically minced into small pieces, and then the small pieces were enzymatically digested in DMEM containing 10% FBS and 250 U/ml type I collagenase under continuous shaking at 37 °C for 5 h. The digested solutions were filtered through a mesh size 70 μm cell strainer to remove leftover undigested tissue fragments then filtered again through a mesh size 40 μm cell strainer to separate stromal like stem cell populations from epithelial gland cells and their aggregates. Isolated endometrial stem cells were then expanded, following culture conditions previously described [22]. Normal human stromal cells (CRL-1947) were obtained from the Korean Cell Line Bank (Seoul, Republic of Korea) and then expanded in 10% FBS and 1% Pen/Strep-containing DMEM. Normal human umbilical vein endothelial cells (HUVECs) were obtained from ATCC (PCS-100-010) and then expanded in EBM-2 culture medium supplemented with EGM-2 single quote kit (Lonza). Normal human macrophages were obtained from ATCC (CRL-9855) and then expanded in 10% FBS and 1% Pen/Strep-containing RPMI 1640. The culture media for individual cell types were changed every 2–3 days depending on their confluency. Elongated and spindle-shaped human myometrial cells were isolated lower segment of myometrial biopsies. Similar to the isolation process of endometrial stem cells, myometrial samples were mechanically minced into small pieces, and then the small pieces were enzymatically digested in DMEM containing 10% FBS and 250 U/ml type I collagenase under continuous shaking at 37 °C for 5 h. The digested solutions were filtered through a mesh size 70 μm cell strainer to remove leftover undigested tissue fragments then filtered again through a mesh size 40 μm cell strainer to separate spindle-shaped human myometrial cell populations from epithelial gland cells and their aggregates. Isolated human myometrial cells were then cultured, following culture conditions previously described by Gargett et al. [23].

### Fabrication procedure of endometrial tissue blocks and assembly sheets encapsulating various endometrial cells

The mixture of biomaterials (collagen and hyaluronic acid) and blood coagulation factors (fibrinogen and thrombin) with approximately 1 × 10^6^ individual endometrial cells were injected into 3D printed casting mold for various tissue blocks or assembly sheets then placed in the vacuum chamber for degassing process. Type I collagen (3 mg/ml) and hyaluronic acid (3 mg/ml) were prepared in DMEM containing 10% FBS and 1% Pen/Strep. For cross-linking the biomaterial mixture without toxicity, two blood coagulation factor [fibrinogen (12.5 mg/ml) and thrombin (1.25 U/ml)] solutions were also prepared in separate growth media. Biomaterial mixture with certain type of endometrial cells and two blood coagulation factor solutions were injected into the casting molds (square, rectangle, or circular shape) and then polymerized them in a room temperature for 1 h. Next, polymerized tissue blocks were effectively released from the fabricating mold by pushing slowly them with a separator. Individually synthesized endometrial tissue blocks were inserted into the multiple empty slots on the prefabricated assembly sheet to form the multi-modular endometrium architecture.

### Analyzing various physical properties of fabricating biomaterial mixture: microstructure, mechanical strength, and rheology

**Microstructure**: The microstructures of fabricating biomaterial mixture was analyzed using a field-emission scanning electron microscopy (SEM) (VP-FE-SEM; EVO®LS10, Carl Zeiss, Germany) at the Korean Basic Science Institute (KBSI, Chuncheon, Korea). Fabricating biomaterial mixture was freeze-dried, and then the samples were coated with an ultra-thin layer (10 nm) of gold/palladium alloy to allow high-resolution imaging using an ion sputterer (Ion Sputter 1010, Hitachi, Japan) at 15 mA discharge current for 30 s. The electron microscope images were obtained at an accelerating voltage of 1.2–1.3 kV following previously described methods [24]. **Mechanical strength**: The mechanical properties of fabricating biomaterial mixture samples (3 mm height and 10 mm diameter) were studied by applying uniaxial compression force with a universal testing machine (QM100S, QMESYS, Gunpo, Korea). A compression force was loaded to samples at a displacement rate of 5 mm/min^− 1^ until the samples broke to estimate the stress levels at failure according to previously described methods [24]. **Rheology**: viscosities of the fabricating biomaterial mixture samples were analyzed by using a Anton Paar MCR 102 rheometer (Anton Paar, zofingen, Switzerland) at 37 °C in both PBS solution and DW; varying the shear rate from 1 s^− 1^ to 20 s^− 1^ allowed analysis of potential rheological behavior during the transitions between shear thinning and shear thickening following previously described methods [24].

### Live & Dead assay for cell viability assessment

Long-term cytocompatibilities of various embedded cells within the biomaterials-based tissue blocks were analyzed by performing a Live & Dead assay (Invitrogen and Cat. No: L3224) after 1, 7, 14, 21, and 28 days of endometrial cell encapsulation according to the manufacturer protocols. Individual tissue blocks were washed three times with DMEM medium without serum, and then a 1 ml assay solutions containing 4 mM calcein AM and 2 mM EthD-1 were added onto each tissue blocks encapsulating various endometrial cells. After 30 min incubation at room temperature, cell viabilities within each tissue blocks were observed using a EVOS FL Cell Imaging System (Thermo fisher scientific, Waltham, USA).

### Analyzing metabolic activities of embedded cells within tissue block by performing CCK-8 assay

Long-term metabolic activities of embedded cells within the biomaterials-based tissue blocks were evaluated with or without estrogen treatment (2 mM) by performing CCK-8 assay (Abbkine and Cat. No: KTC011001) according to the manufacturer protocols. Briefly, each tissue block was incubated with 100 µl/ml of CCK-8 solution in serum-free medium. Cells-embedded tissue blocks were then incubated in the 5% CO_2_ incubator at 37 °C for 4 h. The metabolic activities were measured by absorbance at 450 nm using the microplate reader Soft max pro 5 (Molecular device, San Jose, USA).

### Immunofluorescent staining of embedded cells within tissue blocks

Tissue block samples were fixed for 15 min at room temperature with 4% paraformaldehyde in PBS followed by 2–3 washes with PBS. Embedded cells within tissue blocks were permeabilized with PBS containing 0.4 M glycine and 0.3% Triton X-100, and nonspecific reactions were prevented with 1 h incubation in blocking solution containing 2% normal goat serum (DAKO, Glostrup, Denmark). Immunostaining of each tissue block was performed as described previously [25], using the primary anti-GFP (Invitrogen and Cat. No: V820-20), mCherry (TAKARA and Cat. No:632,524), estrogen receptor (Santa cruz biotechnology, Cat. No.: sc-8005), progesterone receptor (Santa cruz biotechnology, Cat. No.: sc-810), vimentin (BD biosciences, Cat. No.: 550,513), cytokeratin 14 (Santa cruz biotechnology, Cat. No.: sc-53,253), PECAM1 (R&D systems, Cat. No.: BBA7), von Willebrand factor (Abcam, Cat. No.: ab6994), CD11b (Abcam, Cat. No.: ab52478), CD68 (Santa cruz biotechnology, Cat. No.: sc-20,060), myogenin (Abcam, Cat. No.: ab1835), and myo D (Santa cruz biotechnology, Cat. No.: sc-377,460) antibodies. Protein expression patterns of embedded cells within each tissue blocks were examined under a fluorescence microscopy (Zeiss LSM 510 Meta).

### Antibody-pair based cytokine arrays

The cytokine arrays were carried out according to the manufacturer’s methods (Abnova AA0089). Various endometrial cells encapsulating multi-modular tissue assembly or cell-free tissue architecture was incubated in specific culture media for 7days, and then switched from serum-supplemented medium into serum-free media. After additional 48 h in culture, the media were harvested and reacted with the nitrocellulose membranes spotting capture various antibodies overnight at 4 °C. After washing three times with PBS, 2 ml of biotin-conjugated anti-cytokine antibody solutions were added to each nitrocellulose membrane and incubated overnight at 4 °C. The membranes were then washed 3 times with PBS and incubated with diluted HRP-conjugated streptavidin. Chemiluminescent signal detection was used to analysis the expression patterns of various cytokines and growth factors spotted on the nitrocellulose membrane.

### Enzyme-linked immunosorbent assay (ELISA)

Specific levels of PEG_2_ (R&D systems, Cat. No.: KGE004B) or prolactin (R&D systems, Cat. No.: DPRL00) produced and secreted by multi-modular endometrial tissue assembly was analyzed using ELISA kits according to the manufacturer’s protocols. The optical density of each well was then determined using a microplate reader with absorbance wavelength at 450 nm, and PEG_2_ or prolactin concentrations in each tissue sample were evaluated using the absorbance curve. Experiments were carried out in triplicate.

### Analysis of Gene expression Omnibus (GEO) database

Gene Expression Omnibus (GEO) is an international public database repository of high throughput gene expression data accessible through the website (https://www.ncbi.nlm.nih.gov/geo/). The GEO database is created by DNA microarrays, chip sequencing, and genome hybridization arrays [26, 27]. Researchers provide their various experimental information, such as raw data files, experimental designs, sample size, and analysis platform. Experimental or clinical datasets within each sample are additionally categorized according to their various experimental conditions, such as physiological states, treated groups, and disease conditions. These categorized experimental results is presented as a “GEO profiles”, which comprises the dataset title, a diagram describing the expression levels, gene identity annotation, and their ranking across each groups [28]. The expression profiles of seven prominently secreted factors secreted from tissue assembly during menstruation-associated endometrial changes were evaluated according to previously described methods [28].

### Analysis of therapeutic effects of multi-modular tissue assembly on extensive skin defect animal models

All of the animal experiments were carried out according to the approved protocol of Institutional Animal Care and Use Committee (IACUC) (LCDI-2022-0034) of Gachon University. Immunocompromised NSG mice (7 weeks old, female, body weight 15–20 g) were purchased from Jackson Laboratory (Bar Harbor, ME, USA). A Full-thickness excision injury of dorsal skin were created under anesthesia using a biopsy punch device (8 mm diameter) following removal of hair. A transparent film dressing (3 M Tegaderm) was covered to protect the injury sites after topical transplantation of multi-modular tissue assembly or cell-free tissue construct. Before transplantation, embedded cells within multi-modular tissue assembly were labelled with fluorescent GFP or mCherry expressing vector. The mice were housed individually in ventilated cages with sterile conditions. The degree of injured tissue regeneration was analyzed on day 0, day 7, day 14 and 21 day. After sacrifice by CO_2_ exposure, wounded tissue samples were fixed with 10% neutral buffered formalin and used for histopathological analysis (hematoxylin & eosin staining) and immunohistochemistry staining.

### Data presentation and statistical analysis

Statistical analysis was performed using GraphPad Prism version 9.0 (GraphPad Software, San Diego, CA, USA) and two-tailed Student’s t-tests. A p-value of 0.05 or lower was considered to statistically significant (*p < 0.05, **p < 0.005, and ***p < 0.001).

## Results

### Fabricating endometrial tissue blocks (modules) by integrating traditional mold casting and 3D printing techniques

The basic concept of a multimodular tissue assembly recapitulating the multicellular structural complexity of the human endometrium by assembling five different endometrial tissue blocks (modules) is well described in Fig. 1A-D. Individual tissue blocks were fabricated by incorporating certain endometrial cellular components (endometrial stem cells, immune cells, muscle cells, stromal cells, or vascular endothelial cells) and various biomaterials (hyaluronic acid, collagen, and agarose) with nontoxic blood coagulation factors (fibrinogen and thrombin) as natural crosslinking agents. Individual tissue blocks were synthesized by injecting the biomaterial mixture and specific endometrial cell type into the 3D printed fabricating mold and then polymerized. Next, individually synthesized tissue blocks were properly assembled by inserting them into multiple empty slots on the prefabricated assembly sheet to stably form the customizable multimodular endometrial tissue architecture.

To reliably and effectively synthesize tissue blocks, we developed casting molds for individual endometrial tissue blocks using a polylactic acid (PLA)-based 3D printing technique. PLA was chosen due to its cost, mechanical strength, printability, and availability [29]. The development procedure for fabricating molds by PLA-based 3D printing is illustrated in Fig. 2A and C. Three different types of fabricated PLA molds (50 × 30 × 2 mm^3^) were 3D printed, and each mold could produce 15 endometrial tissue blocks at once: (i) square block mold (50 × 30 × 2 mm^3^), (ii) rectangle block mold (50 × 30 × 2 mm^3^), and (iii) circular block mold (50 × 30 × 2 mm^3^) (Fig. 2B). In addition, to effectively separate the polymerized tissue blocks from the casting mold without damage, we developed a separator that can properly release the fabricated tissue block from the molds by pushing them using PLA-based 3D printing (Fig. 2B).

In addition, one of the major challenges of bottom-up tissue creation is to assemble individual tissue blocks into stable tissue architectures with robust mechanical strength for withstanding clinical implantation and exerting sufficient therapeutic effects [30]. In this study, we therefore assembled individual tissue blocks by inserting them into a biomaterial-based assembly sheet, which has multiple empty slots with the same size and shape as individual tissue blocks, as illustrated in Fig. 2C. Thus, the casting molds for three different types of assembly sheets (20 × 20 × 5 mm^3^), which contain multiple slots for individual endometrial tissue blocks, were also fabricated using PLA-based 3D printing (Fig. 2D).

### Establishment of optimal biomaterial combinations for fabricating endometrial tissue blocks and the analysis of their various mechanical characteristics

Another challenge of developing biomaterial-based tissue architectures is properly sustaining the mechanical strength to sufficiently support better survival, attachment, and subsequent therapeutic efficacy of embedded cells [31, 32]. Among various natural polymers, hyaluronic acid and collagen, as the primary structural elements of the extracellular matrix (ECM), have been widely utilized in many tissue engineering fields because of their high solubility in water, favorable biocompatibility, and biodegradability [33–35]. However, their mechanical strength is not sufficient to properly reflect the physical properties of a wide range of normal tissues. Thus, to reinforce their mechanical strength without toxicity, we incorporated blood coagulation factors (fibrinogen and thrombin) as natural crosslinking agents into the biomaterial mixture. We established optimal biomaterial combinations for individual tissue blocks and then evaluated their various physical characteristics, such as porous morphology, mechanical strength, viscosity, and wettability.

**Fig. 3:**
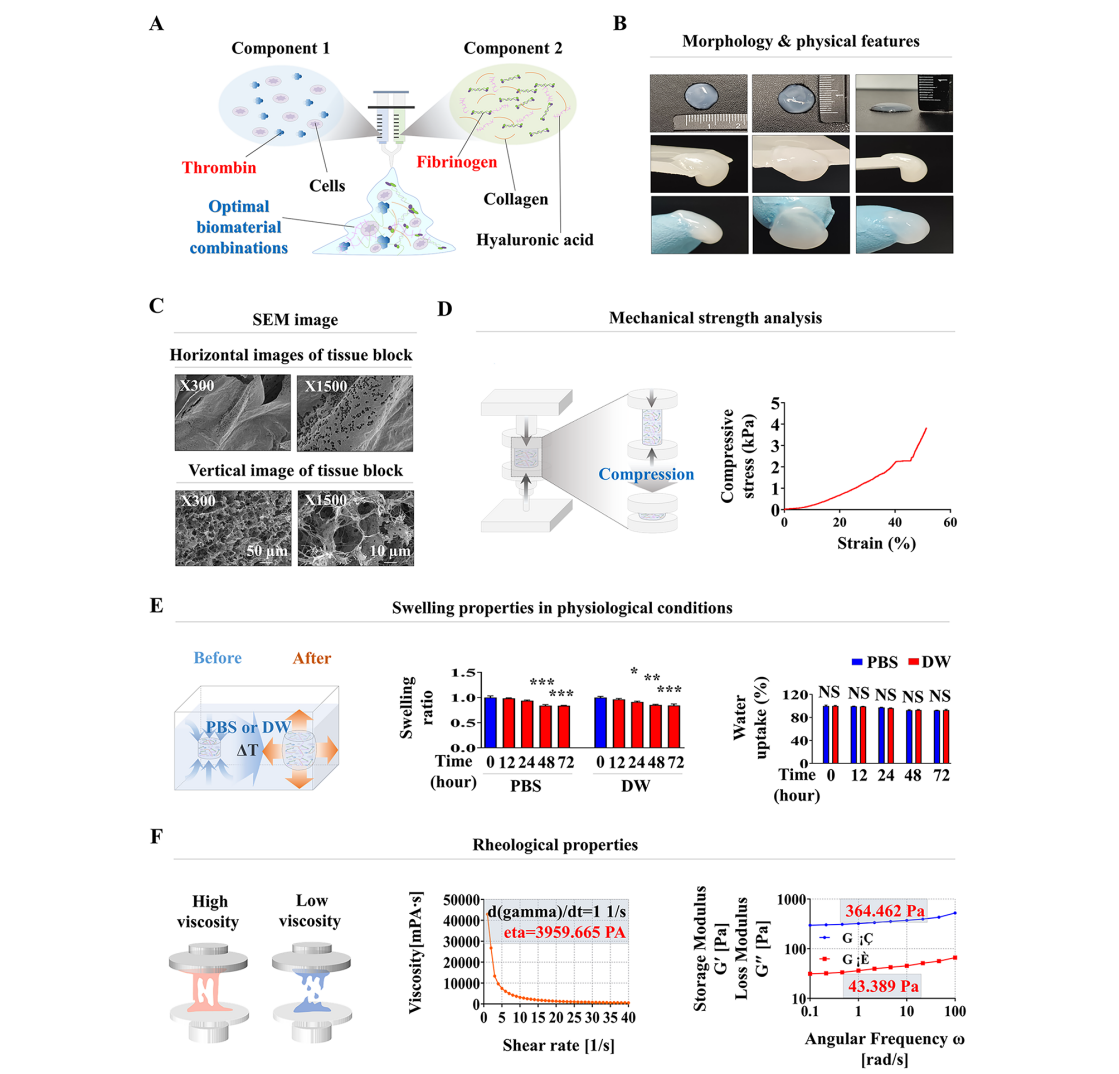
**Analyzing various physical properties of the fabricating biomaterial mixture for tissue block synthesis.** The mixture of two biomaterials (collagen and hyaluronic acid) was properly polymerized by adding two blood clotting factors (thrombin and fibrinogen) as a natural crosslinker agent, which markedly reinforced the mechanical properties of the tissue block fabricating natural polymers **(A)**. The optimal biomaterial combinations, which can encapsulate various types of endometrial cells, appeared to retain water and had a soft texture with white color **(B)**. Vertical and horizontal scanning electron microscopy (SEM) images of the fabricated biomaterials showed an irregularly distributed and highly porous microstructure with a pore diameter between 10 and 50 μm throughout the biomaterial mixture **(C)**. Their mechanical strength was analyzed using the stress–strain plot under uniaxial compression (pressing) force with a material testing machine. An increasing force was gradually applied at a loading rate of 5 mm/min^− 1^ until the sample mechanically failed to analyze the compressive strength according to our previously established conditions **(D)**. For evaluation of the hydration-induced swelling behavior of the biomaterial mixture, samples were hydrated in PBS (pH 7.4) or DW at 37 °C with gentle shaking. PBS or DW was completely removed from biomaterials, and then, their water absorption capacities were calculated by weighing before and after soaking **(E)**. The rheological behavior of the biomaterial mixture was estimated as described in the methods section, and applying different shear rates ranging from 1 s^− 1^ to 40 s^− 1^ allowed the measurement of their shear thinning and shear thickening potential **(F)**. Significant differences are presented. *p < 0.05, **p < 0.005, and ***p < 0.001 (two-sample t-test)

The synthetic procedure of optimal biomaterial combinations for endometrial tissue block by integrating biomaterial mixture (collagen and hyaluronic acid) and blood coagulation factors (fibrinogen and thrombin) is illustrated in Fig. 3A, and the optimally combined biomaterials showed very soft texture with white color (Fig. 3B). The microstructural properties of fabricating biomaterials for endometrial tissue blocks were observed through scanning electron microscopy (SEM). The morphological analysis showed homogeneously distributed micropore structures throughout the fabricated biomaterials. These pore sizes can be changed by adjusting the amount of the core polymerizing backbone fiber: as the amount of hyaluronic acid increases, the pore size decreases. These micropore structures (approximately 10–50 μm in diameter) were mediated through the stable polymerization and successive crosslinking processes of combined biomaterials (hyaluronic acid, collagen, and coagulation factors) (Fig. 3C).

Next, we analyzed mechanical strength to obtain physical information about biomaterial-based tissue blocks. The results revealed that the mechanical properties of fabricating biomaterials were approximately 4 kPa and showed similar physical strength to actual endometrial tissue (Fig. 3D). In addition, swelling analysis of fabricating biomaterials under physiological conditions (37 °C in both PBS solution and DW) indicated that their shape and size were not dramatically affected by wet conditions under physiological conditions (Fig. 3E).

Moreover, the rheological behavior (viscosity) of fabricating biomaterials is highly correlated with the physical characteristics of tissue blocks and the biological prosperities of embedded cells. For instance, low biomaterial viscosity results in poor structural integrity, but the viabilities of embedded cells are relatively high [36]. Thus, suitable rheological behavior that is highly associated with multiple cellular functions, such as cell adhesion, viability, and migration [37], needs to be properly considered. The rheological behavior of fabricating biomaterials was analyzed by measuring the viscosity over the shear rate. The viscosity of fabricating biomaterials decreased from approximately 4000 to 0 Pa-sec as the shear rate increased stepwise from 1/s to 40/s (Fig. 3F). These results suggested that the fabricated biomaterials can be suitable platforms to encapsulate various endometrial tissue constituent cells.

### Fabrication of endometrial tissue blocks and assembly sheets by injecting biomaterials and endometrial cells into a 3D printed PLA casting mold

For fabrication of individual tissue blocks encapsulating various endometrial cell types, five different human endometrial tissue constituting cells were freshly isolated or obtained. First, human endometrial stem cells were freshly isolated from surgical specimens and cultured as previously reported [22] (Suppl. Figure 1 A), and several cellular properties were evaluated using flow cytometry with various putative positive and negative markers, such as CD34, CD44, CD45, CD73, CD105, CD140b, CD146, and SUSD2 (Suppl. Figure 1B). Their pluripotent potential to differentiate into multiple cell lineages was also examined by inducing differentiation into adipocytes and osteoblasts (Suppl. Figure 1 C). Second, we performed in vitro expansion of human stromal cells obtained from skin specimens. Phenotypically, these cells were characterized by elongated fibroblast-like shapes (Suppl. Figure 2 A) and positive immunostaining for vimentin and low levels of cytokeratin 14 (CK14) expression (Suppl. Figure 2B). Third, HUVECs, endothelial cells derived from the human umbilical cord, are one of the most commonly used cellular models for vascular formation research in vitro [38]. Therefore, we employed these cells as a cell model for endometrial vascular formation. HUVECs were phenotypically characterized by polygonal shape (Suppl. Figure 3 A) and positive immunostaining for PECAM1 and von Willebrand factor (vWF) (Suppl. Figure 3B). Fourth, macrophages are known to be involved in various physiological processes of endometrial development by interacting with other cell types [39, 40]. Thus, we used human macrophages as an immune cell model of endometrial regeneration. These cells were morphologically characterized by their round shape (Suppl. Figure 4 A) and positive immunostaining for the specific surface markers CD11b and CD68 (Suppl. Figure 4B). Finally, uterine tissue-derived human myometrial cells, the main cell type constituting the outside muscle layer of the endometrium, were also characterized by their spindle shape (Suppl. Figure 5 A) and positive immunostaining for MyoD and myogenin (Suppl. Figure 5B).

**Fig. 4:**
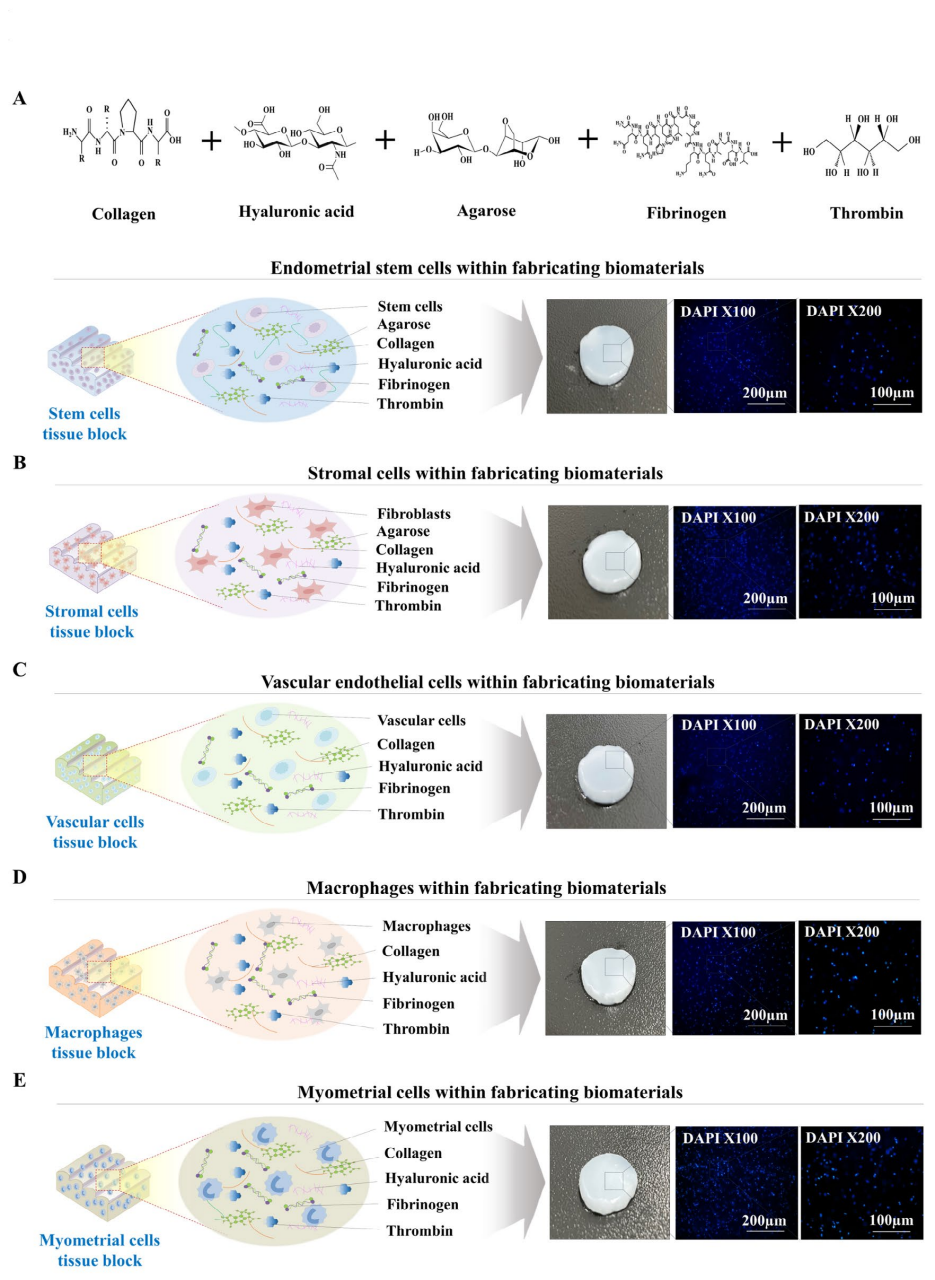
**Various encapsulated endometrial cells are distributed homogeneously throughout the fabricated biomaterials.** For analysis of the distribution patterns of the embedded cells within individual endometrial tissue blocks, samples were incubated in optimized culture medium for each cell type and then incubated in nuclear-specific dye DAPI solution. Each sample was observed using a fluorescence microscope with appropriate filters. Various embedded endometrial cellular components within individual tissue blocks, such as endometrial stem cells **(A)**, stromal cells **(B)**, vessel cells **(C)**, macrophages **(D)**, and myometrial cells **(E)**, were labeled with DAPI (blue color). The labeled cells were homogeneously distributed throughout biomaterial-based individual tissue blocks

**Fig. 5:**
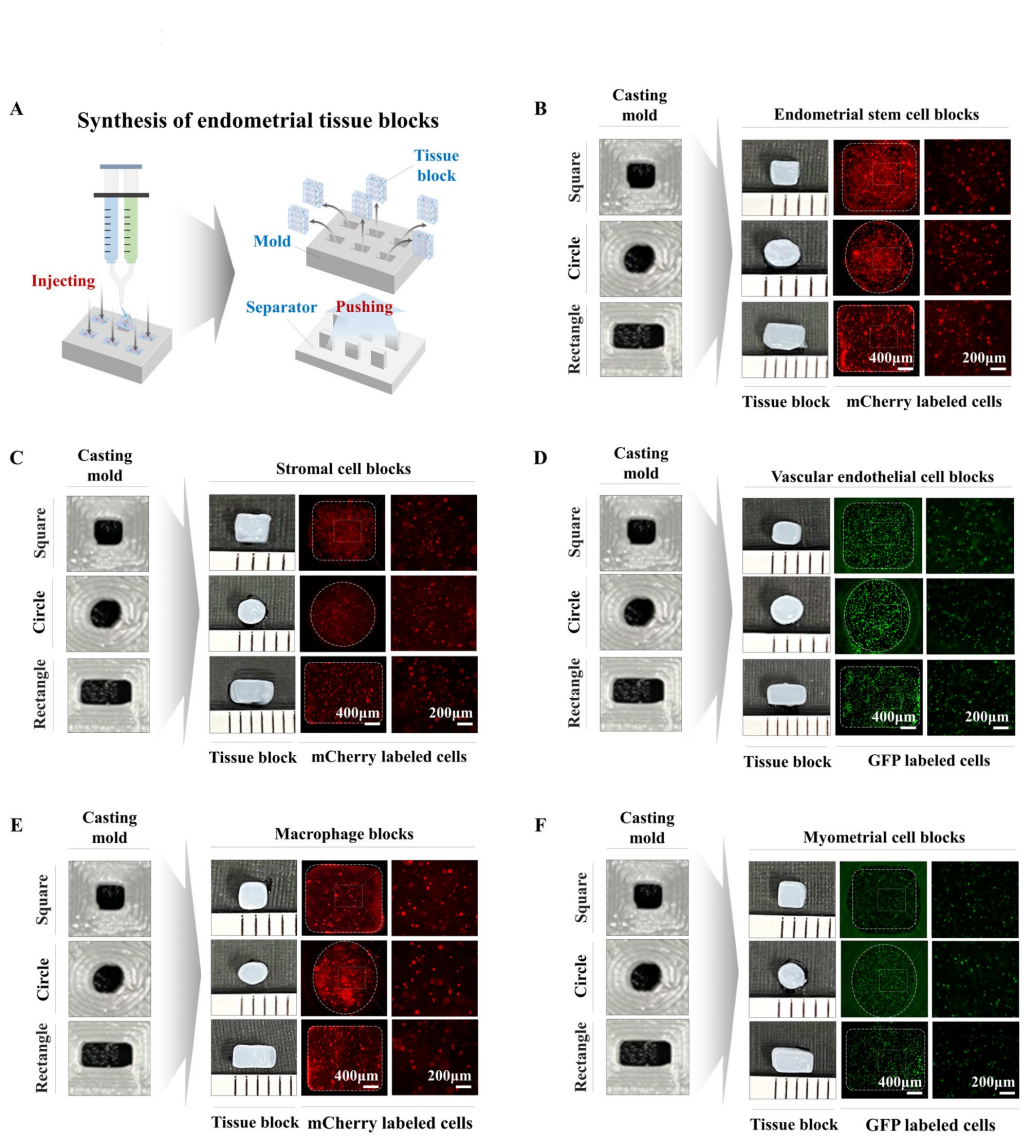
**Synthesis of individual endometrial tissue blocks encapsulating various types of endometrial cells labeled with fluorescent dyes.** The fabricated biomaterials encapsulating various endometrial cellular components were injected into casting molds for three different block types (square, rectangle, and circular shape) and then polymerized at room temperature. Next, polymerized tissue blocks were effectively released from the casting molds by pushing them with a separator **(A)**. Each embedded endometrial cellular component was labeled with GFP (green fluorescent protein) or mCherry (red fluorescent protein) to track their incorporation into the injured sites when they were transplanted in vivo. Three different types (square, rectangle, and circular shape) of tissue blocks encapsulating specific endometrial cellular components, such as endometrial stem cells **(B)**, stromal cells **(C)**, vessel cells **(D)**, macrophages **(E)**, and myometrial cells **(F)**, were properly fabricated, and labeled cells were homogenously distributed throughout individual tissue blocks

**Fig. 6:**
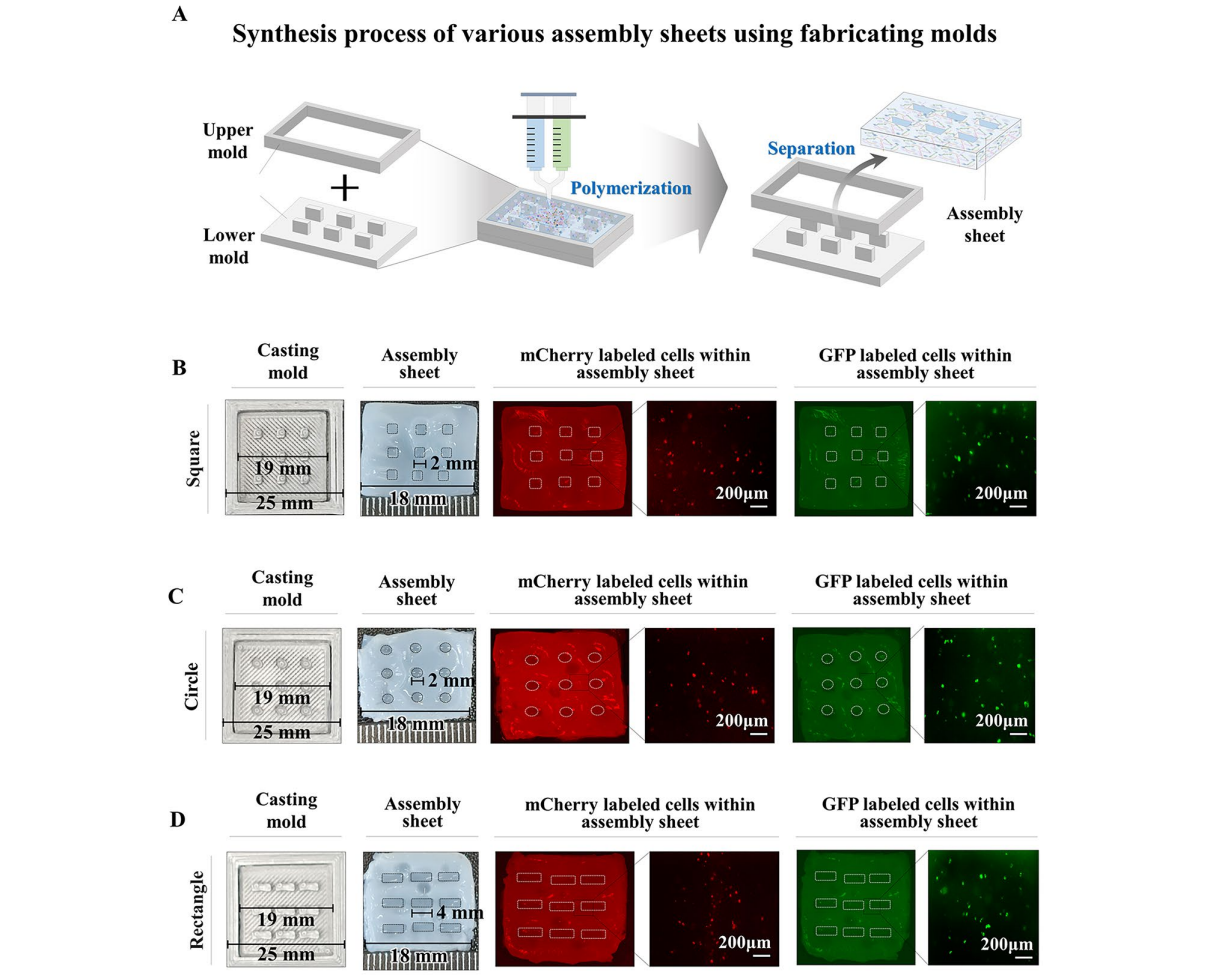
**Synthesis of various assembly sheets encapsulating various endometrial cells labeled with fluorescent dyes.** The fabricated biomaterials encapsulating various endometrial cellular components were injected into casting molds for three different types (square, rectangle, and circular shape) of assembly sheets and then polymerized at room temperature. Next, polymerized assembly sheets were effectively released from the molds by separating the upper and lower molds **(A)**. Each endometrial cell type was labeled with GFP (green fluorescent protein) or mCherry (red fluorescent protein) to track their incorporation into the injured sites when they were transplanted in vivo. Three different types, square **(B)**, rectangle **(C)**, or circular **(D)** shape assembly sheets encapsulating various endometrial cell types, were properly fabricated, and labeled cells were homogenously distributed throughout individual assembly sheets

The spatially uniform cellular distribution in bioengineered tissue is one of the key factors that affects overall functional features and ultimately their successful therapeutic effects [41]. Indeed, we observed that the five different embedded endometrial cell types (endometrial stem cells, immune cells, myometrial cells, stromal cells, or vascular endothelial cells) were homogenously distributed throughout fabricating biomaterials (Fig. 4A-E). In addition, to track the transplanted multimodular endometrial tissue assembly in vivo, we labeled various encapsulated cells with GFP (green fluorescent protein) or mCherry (red fluorescent protein) (Suppl. Figure 6A-E). To produce individual endometrial tissue blocks and assembly sheets effectively, we injected fabricating biomaterials with certain types of endometrial cells into the three different types of casting molds (square, rectangle, or circular shape) and then polymerized them at room temperature. Next, polymerized tissue blocks (Fig. 5A) and assembly sheets (Fig. 6A) were effectively released from the casting mold by pushing them with a separator. The five different endometrial tissue blocks (Fig. 5B-F) and three kinds of assembly sheets (Fig. 6B-D) appropriately sustained their own morphological shape, and various types of embedded endometrial cells, constitutively labeled with fluorescent GFP (green) or mCherry (red), were homogenously distributed throughout individual tissue blocks.

### Cytocompatibility analysis of encapsulated cells within individual endometrial tissue blocks: cell viabilities, metabolic activities, and molecular characteristics

**Fig. 7:**
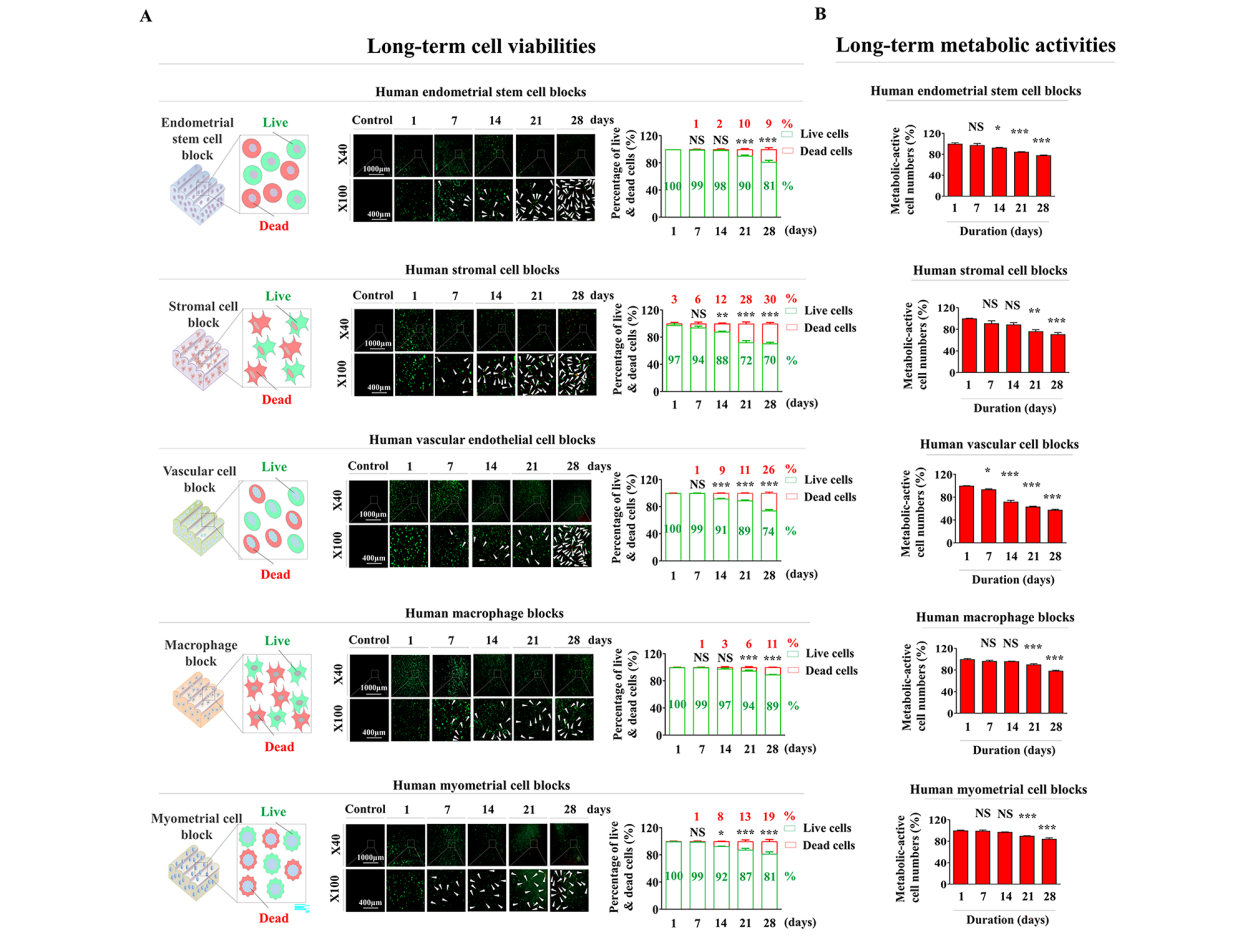
**Analysis of the long-term cytocompatibility of various embedded endometrial cells within each tissue block.** Individual tissue blocks embedded with individual endometrial cells were incubated in their specific culture media for 1, 7, 14, 21, and 28 days after cell encapsulation, and then, the assay solution was added to each sample. Cell viabilities within individual tissue blocks were assessed using a fluorescence microscope. At least 70% of embedded cells within the individual tissue blocks remained viable 28 days after encapsulation (green fluorescence indicates living cells, and red fluorescence indicates dead cells) **(A)**. Similarly, individual tissue blocks were incubated in their specific culture media for 1, 7, 14, 21, and 28 days after cell encapsulation, and then each tissue block sample was additionally incubated with CCK-8 solution in serum-free medium for 48 h **(B)**. All experiments were performed in triplicate. Significant differences are presented. *p < 0.05, **p < 0.005, and ***p < 0.001 (two-sample t-test)

Although fabricating biomaterials can result in ideal mechanical characteristics for tissue repair, other technical challenges still remain to be addressed. These challenges are mostly associated with the cytocompatibility maintenance of embedded cells within the tissue blocks. We thus analyzed the cell viabilities and multiple cellular functions of various encapsulated endometrial cell types within individual tissue blocks in vitro. Their long-term cell viabilities were evaluated using live & dead assays at several time points (24 h, 7, 14, 21, 28 days). Although the overall cell viabilities of various encapsulated cells within individual tissue blocks were gradually reduced over time, most of them (approximately 70–89%) were still alive up to 28 days (Fig. 7A). In addition, their metabolic activities during long-term cultivation in individual tissue blocks were evaluated at the same time points using CCK-8 assays after cell encapsulation. Consistently, even though their metabolic functions were also continuously reduced over time, at least 80% of the embedded cells remained metabolically active (Fig. 7B).

**Fig. 8:**
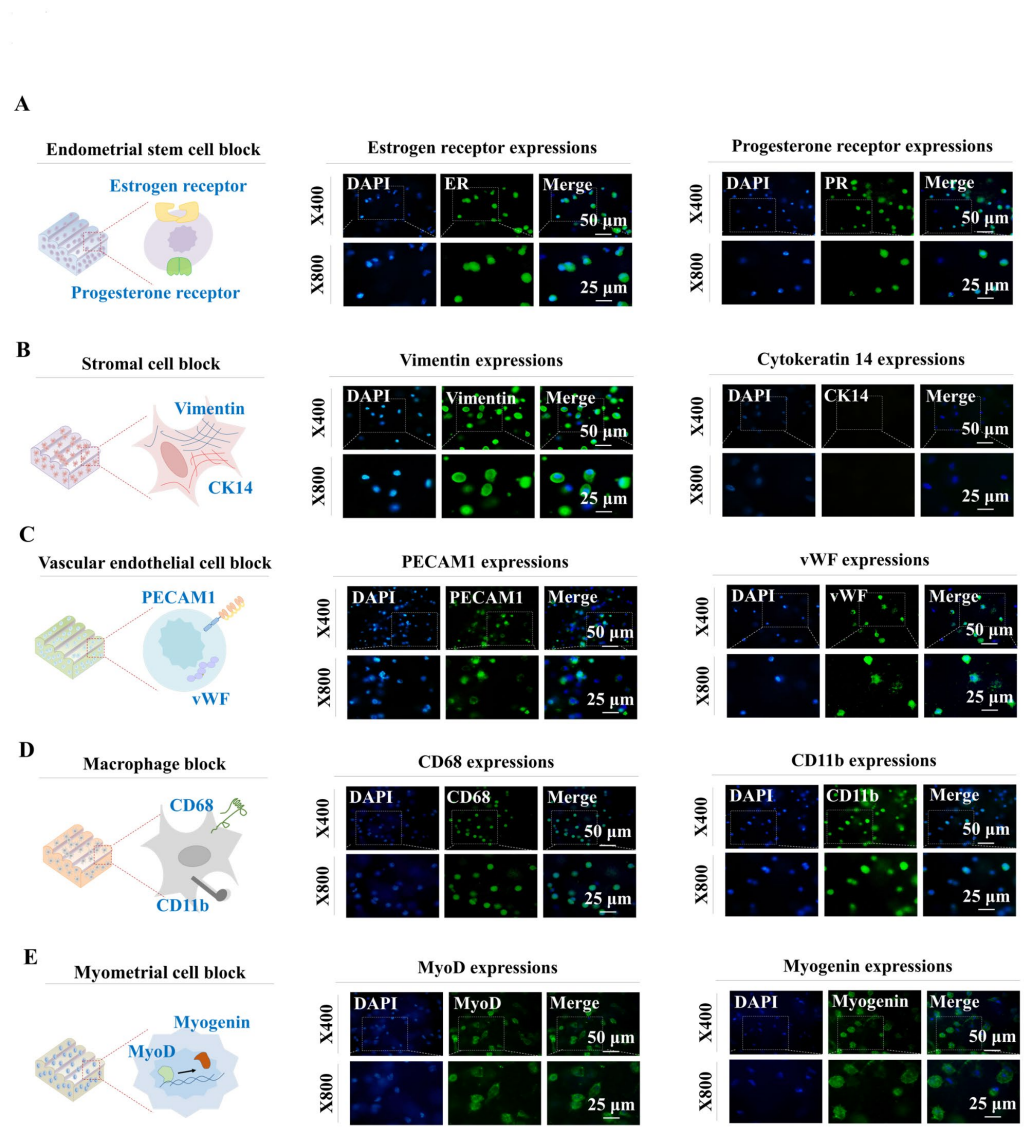
**Evaluating the maintenance of tissue-specific characteristics of embedded cells within individual endometrial tissue blocks.** For evaluation of whether the encapsulated cells within individual tissue blocks properly maintained their own specific cellular characteristics, block samples were incubated in specific culture media for 7 days, and the expression patterns of their typical biomarkers were assessed: endometrial stem cells were stained for estrogen and progesterone receptors **(A)**, stromal cells were stained for cytokeratin (CK) 14 and vimentin **(B)**, vessel cells were stained for PECAM1 and vWF **(C)**, macrophages were stained for CD11b and CD68 **(D)**, and myometrial cells were stained for myogenin and Myo D **(E)**. All experiments were performed in triplicate. DAPI staining was used to label the nuclei within each field

In addition, individual tissue blocks encapsulating certain types of endometrial cells were further evaluated to determine whether each encapsulated cell can properly maintain its own cellular properties within the tissue blocks using their specific surface markers 14 days after encapsulation. The functions of endometrial tissue are closely controlled by the reproductive steroid hormones estrogen and progesterone during menstruation [42]. Thus, we analyzed whether the embedded endometrial stem cells within the tissue blocks properly expressed specific receptors (ER and PR) for those hormones (Fig. 8A). Stromal cells are known to be CK14 negative, whereas they are stained positively with vimentin [43]. Indeed, the stromal cells within the corresponding tissue blocks were strongly positive for vimentin, while CK14 had weak staining (Fig. 8B). PECAM1 and vWF, well-known biomarkers for vessel cells [44], were strongly expressed in the vascular endothelial cells within corresponding tissue blocks (Fig. 8C). In addition, the widely used macrophage-specific biomarkers CD11b and CD68 [45] were highly expressed in the macrophage tissue module (Fig. 8D). The typical myogenic markers MyoD and myogenin [46] were properly expressed in myometrial tissue blocks (Fig. 8E).

**Fig. 9:**
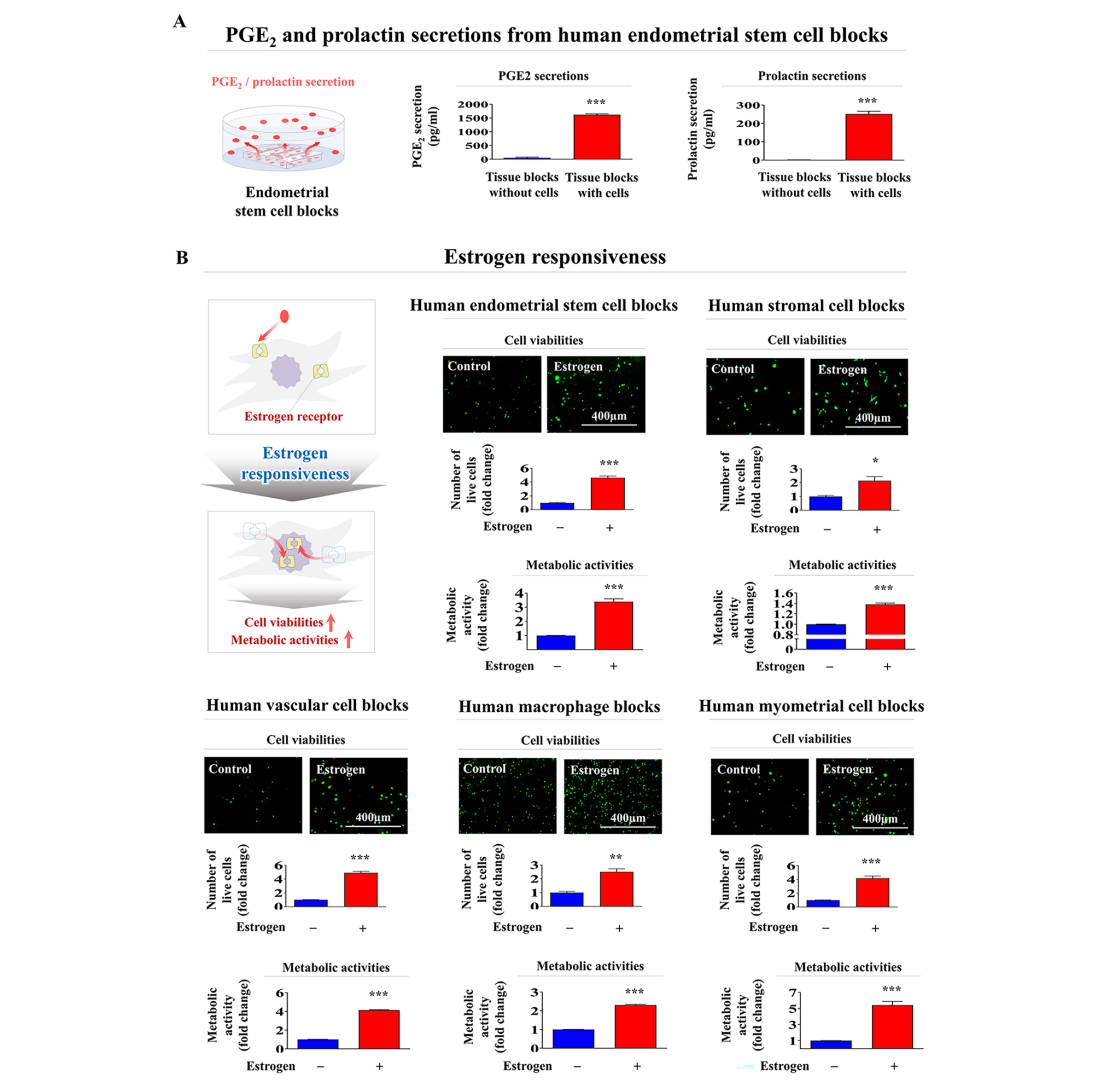
**Functional evaluation of various endometrial encapsulating tissue blocks: reproductive endocrine factor secretion and steroid hormone responsiveness.** For analysis of whether the embedded endometrial stem cells within a tissue block properly secrete their own reproductive endocrine factor, a cell-encapsulating block or cell-free block was incubated in specific culture media for 7 days and then switched from serum-supplemented medium into serum-free media. After an additional 48 h in culture, the media were harvested, and PGE_2_ and prolactin secretion was measured by ELISAs **(A)**. For analysis of the responsiveness of each endometrial tissue block to the steroid hormone estrogen, the cell viability and metabolic activity of embedded cells within individual tissue blocks were assessed by performing live & dead assays and CCK-8 assays, respectively. Various endometrial tissue blocks were incubated in specific culture media for 7 days with or without estrogen exposure, and then, the live & dead assay agents were added to the block samples. Cell viabilities within individual tissue blocks were assessed using a fluorescence microscope. In addition, to assess their metabolic activities, various endometrial tissue blocks were incubated in specific culture media for 7 days with or without estrogen exposure and then switched from serum-supplemented medium to serum-free media. The block samples were additionally cultured for 48 h with CCK-8 solution in serum-free media **(B)**. All experiments were performed in triplicate. Significant differences are presented. *p < 0.05, **p < 0.005, and ***p < 0.001 (two-sample t-test)

The secretion of PGE2 [47] and prolactin [48] from endometrial tissue is one of the key regulatory elements of endometrial receptivity and subsequent embryo implantation. Therefore, we assessed whether PGE2 and prolactin are actively produced and secreted from endometrial stem cell blocks using ELISAs. Consistently, our endometrial stem cell blocks significantly secreted both PGE2 and prolactin into the culture media (Fig. 9A). In addition, steroid hormone responsiveness is one of the critical factors in evaluating whether a fabricated endometrial tissue block functions properly [42, 49, 50]. We therefore analyzed the responsiveness of individual endometrial tissue blocks to the major steroid hormone estrogen by assessing the cell viabilities and metabolic activities with or without estrogen exposure. Importantly, both cell viabilities and metabolic activities of the embedded cells within individual endometrial tissue blocks were markedly enhanced by estrogen exposure (Fig. 9B). These results suggest that the various embedded cells within various endometrial tissue blocks effectively maintain their own cellular properties and functions in response to steroid hormones.

### Development of a multimodular endometrial tissue architecture by assembling individually fabricated tissue blocks and analyzing its functional properties

**Fig. 10:**
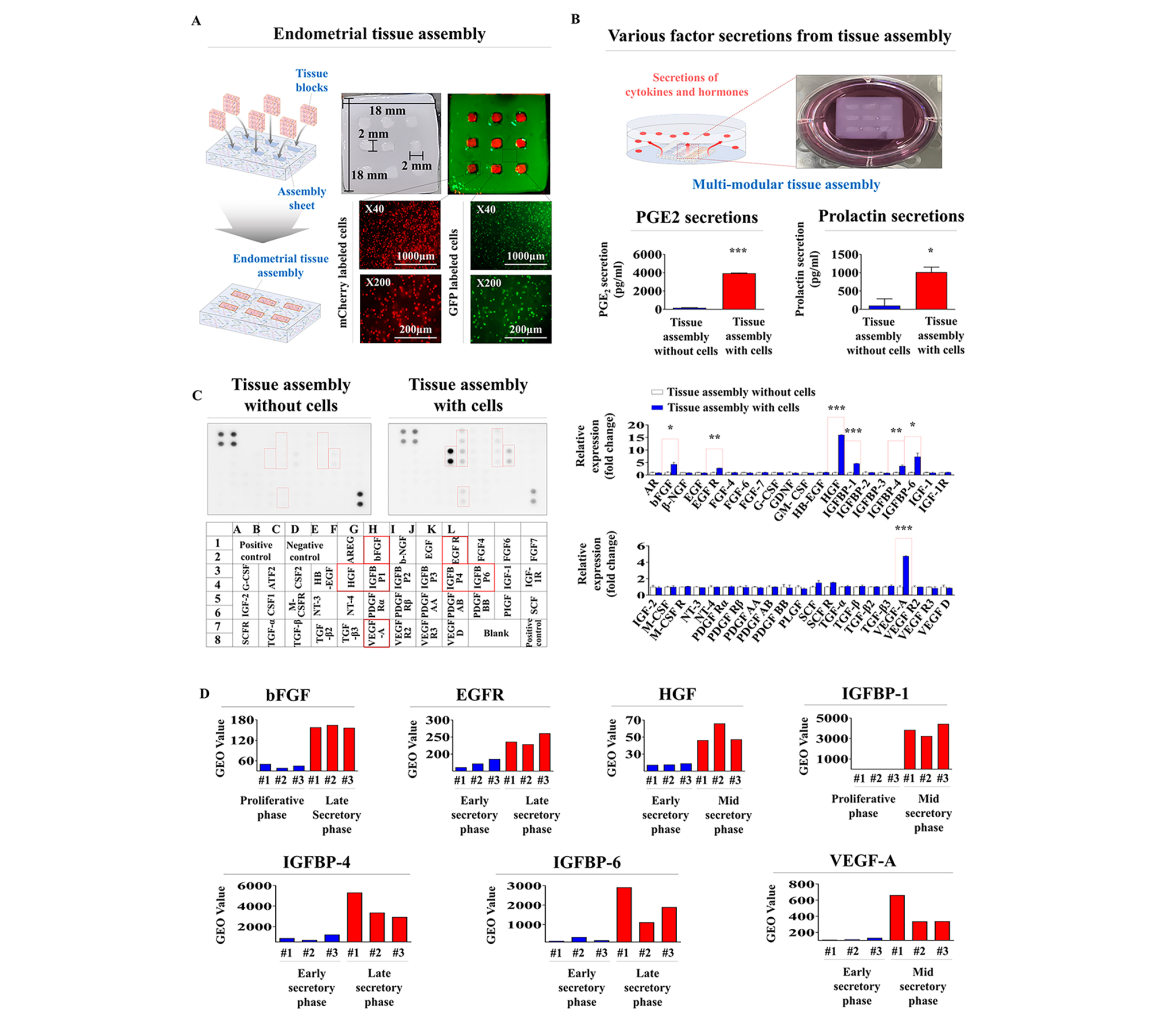
**Functional analysis of multimodular endometrial tissue assembly** in vitro: **reproductive endocrine factor secretion and hormone responsiveness.** The stably assembled multimodular endometrial tissue architecture was archived by inserting individually synthesized tissue blocks into multiple empty slots on the prefabricated assembly sheet. Embedded cells within individual endometrial tissue blocks were labeled with GFP (green fluorescent protein) or mCherry (red fluorescent protein) to track their incorporation into the injured sites when they were transplanted in vivo**(A)**. For analysis of whether the multimodular endometrial tissue assembly properly produces and secretes its own reproductive endocrine factors (PGE_2_ and prolactin), a cell-encapsulating tissue assembly or cell-free tissue architecture was incubated in specific culture media for 7 days and then switched from serum-supplemented medium into serum-free media. After an additional 48 h in culture, the media were harvested, and PGE_2_ and prolactin secretion was evaluated by ELISAs **(B)**. Cell-embedded tissue assembly or cell-free tissue architecture was incubated in specific culture media for 7 days and then switched from serum-supplemented medium to serum-free media. After an additional 48 h in culture, the media were harvested, and the secretion patterns of various growth factors or cytokines were analyzed by performing antibody pair-based cytokine arrays. The membrane was printed with antibodies against forty different growth factors, their receptors, and cytokines **(C)**. The Gene Expression Omnibus (GEO) database, a public data repository for high-throughput gene expression, was also analyzed to further evaluate the correlations between the eleven secreted prominent factors (basic FGF, EGFR, HGF, IGFBP-1, IGFBP-4, IGFBP-6, and VEGF-A) and various menstruation-associated endometrial changes **(D)**. Significant differences are presented. *p < 0.05, **p < 0.005, and ***p < 0.001 (two-sample t-test)

The previous results (Figs. 2, 3, 4, 5, 6, 7, 8 and 9) indicate that individually synthesized endometrial tissue blocks exert their own functions relatively well. Therefore, as a next step, to mimic the multicellular complex structure of endometrial tissue, we developed a customizable multimodular endometrial tissue architecture by assembling individually fabricated endometrial tissue blocks based on patient-specific tissue microenvironments or injured tissue requirements (Fig. 10A). Briefly, the multimodular endometrial tissue assembly was archived by inserting individually synthesized tissue blocks into multiple empty slots on the prefabricated assembly sheet, which also encapsulates various endometrial cells (Fig. 10A). We evaluated whether PGE2 and prolactin are produced and actively secreted from the multimodular endometrial tissue assembly using ELISAs. Importantly, the multimodular endometrial tissue assembly markedly secreted both PGE2 and prolactin into the culture media compared to a cell-free tissue architecture (Fig. 10B). In addition, multiple cytokines or growth factors are known to be produced from the endometrial tissue to support embryo implantation and subsequent pregnancy [51]. Thus, we evaluated whether various endometrial function-associated growth factors or cytokines are actively produced and released from the multimodular endometrial tissue assembly using antibody pair-based cytokine arrays. We observed several changes in the secretion of forty different factors or cytokines from the multimodular endometrial tissue assembly. Among the evaluated factors, seven prominent factors, namely, basic fibroblast growth factor (bFGF), epidermal growth factor receptor (EGFR), hepatocyte growth factor (HGF), insulin-like growth factor-binding protein 1, 4, and 6 (IGFBP-1, 4 and 6), and vascular endothelial growth factor A (VEGF-A), were significantly secreted from the multimodular endometrial tissue assembly, whereas other protein factors were observed to have only slight changes in their secretions (Fig. 10C). These results indicate that the multimodular endometrial tissue assembly shows similar behavior to endometrial tissue by producing various biologically active factors. In addition, we analyzed the GEO database, a public data repository for high-throughput gene expression, to investigate the correlations between endometrial functions and seven prominently secreted factors from the multimodular endometrial tissue assembly. The GEO results revealed that these prominently secreted factors are clearly correlated with menstruation-associated endometrial changes (Fig. 10D). We further analyzed the activation status of various genes and their associated signaling networks using Ingenuity Pathway Analysis (IPA) to further investigate whether these prominently secreted factors are related to various endometrial functions. Positive regulators of HGF, such as TGFß1, EGF, and IGF-I, are activated during endometrial angiogenesis (Suppl. Figure 7 A). Positive regulators of IGFBP6, such as IL-6, OSM and TNF, are activated in inflammatory factor-exposed endometrium (Suppl. Figure 7B). Negative regulators of VEGF, such as TGFß, IL-1ß and ERK1/2, are inhibited during endometrial angiogenesis (Suppl. Figure 7 C). Negative regulators of IGFBP4, such as NOTCH1, TNF, and GATA6, are activated or inhibited during endometrial angiogenesis (Suppl. Figure 7D).

### Analysis of biodegradation rates of the multimodular endometrial tissue assembly in vivo and its regenerative capacity after transplantation

**Fig. 11:**
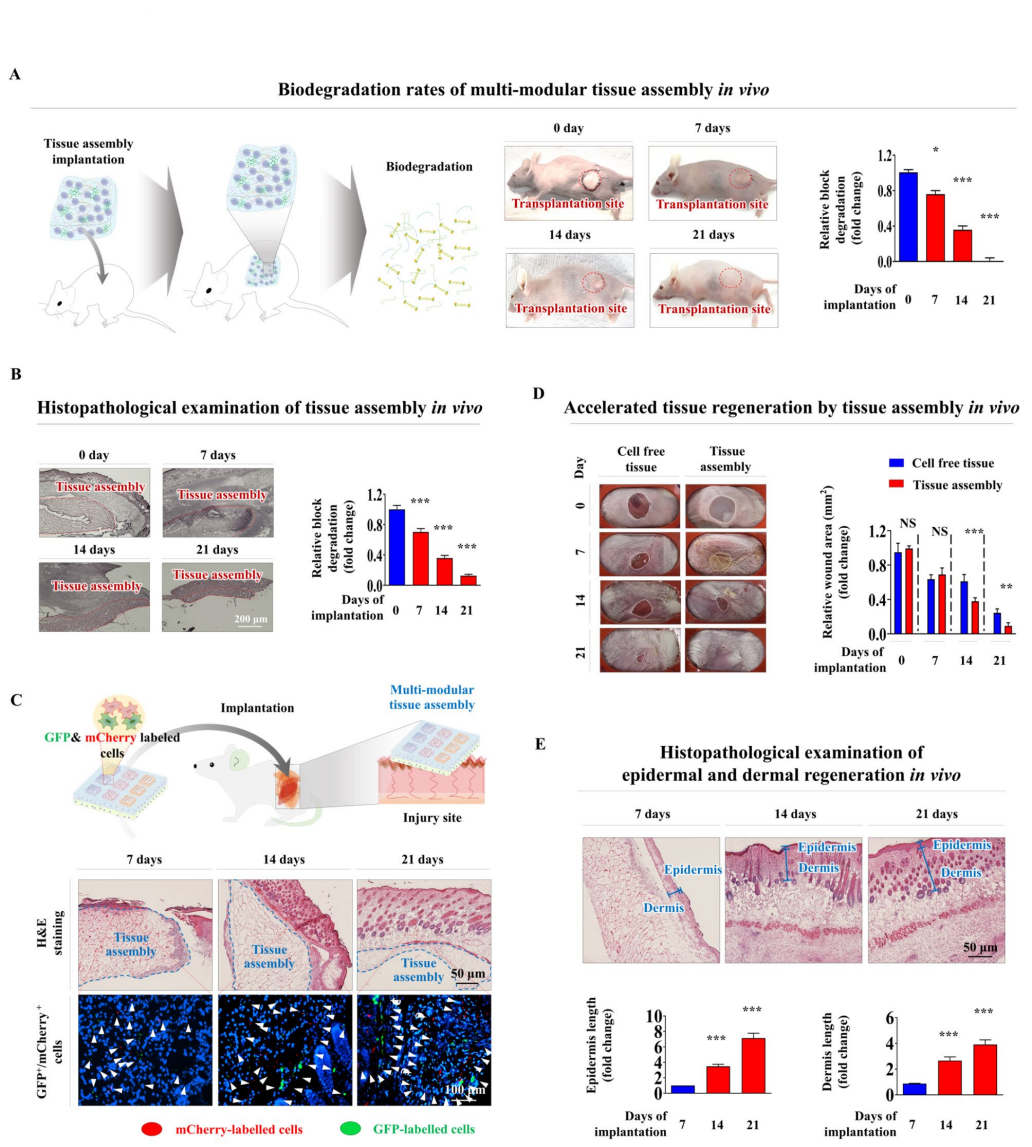
**Functional evaluation of multimodular tissue assembly**in vivo: **biodegradation rates and regenerative capacities.** After transplantation of the multimodular tissue assembly into the abdominal region of mice, we evaluated its biodegradation rates in vivo at 0 days, 7 days, 14 days, and 21 days by assessing its gradually decreased volumes at the transplant sites **(A)** and performing histopathological examination **(B)**. Various embedded cells within the multimodular tissue assembly were labeled with mCherry (red fluorescent protein) or GFP (green fluorescent protein) and then transplanted directly into 7-week-old immunodeficient NSG mice with severe tissue injury **(C)**. Scar formation was then monitored over the subsequent 21 days. The multimodular tissue assembly-implanted wounds showed resurfacing of over 90% of the initial wound area on Day 21 after injury **(D)**. Regenerated tissues were collected and subjected to histopathological examination using hematoxylin and eosin (H&E) staining. Histopathological analysis of wound sites showed that the multimodular tissue assembly-implanted mice revealed significant increases in epidermal and dermal thickness compared to the mice implanted with the cell-free tissue architecture at Days 7, 14, and 21 **(E)**. Significant differences are presented. *p < 0.05, **p < 0.005, and ***p < 0.001 (two-sample t-test)

One of the essential factors to consider before the clinical application of the multimodular endometrial tissue architecture is its appropriate biodegradation potential, which exhibits sufficient structural stability in vivo to exert sufficient therapeutic effects without causing an inflammatory response and toxicity at the site of transplantation. Therefore, after transplantation of endometrial tissue assembly into the abdominal region of mice, we evaluated the biodegradation rates of the multimodular endometrial tissue assembly in vivo by assessing its gradually decreased volumes at the transplant sites and performing histopathological examination (Fig. 11A). Importantly, the transplanted endometrial tissue assembly was gradually degraded and completely disappeared at the transplantation site after 21 days without causing any inflammatory response or toxicity (Fig. 11B). In addition, our in vitro results suggest that the multimodular tissue assembly may serve as an alternative therapeutic option for patients with severe tissue injury. Therefore, a multimodular tissue assembly encapsulating various labeled endometrial cells with GFP (green color) or mCherry (red color) was properly transplanted into the tissue-injured mouse model. Importantly, we observed that various encapsulated cells within the transplanted multimodular endometrial tissue assembly were successfully incorporated into the injury sites (Fig. 11C). In addition, the multimodular tissue assembly-implanted groups exhibited largely accelerated tissue regeneration with minimal scar formation (Fig. 11D). Consistently, histopathological examination revealed that severe degenerative changes with the loss of epidermis and dermis layers were remarkably relieved by the transplantation of the multimodular tissue assembly compared with cell-free tissue architecture (Fig. 11E). Our results suggest that the multimodular endometrial tissue assembly provided a proper 3D microenvironment for enhanced survival and proper tissue incorporation of various encapsulated cells and thus subsequent regeneration of injured tissue in vitro.

## Discussion

In tissue engineering, biomaterial-based porous scaffolds are useful for the regeneration of injured tissue because they can be relatively easily shaped to mimic the structural support and size of certain tissues [52, 53]. However, most traditional single biomaterial-based tissue scaffolds need to overcome several patient-specific parameter-associated challenges, such as the anatomy of the defective area and 3D architectural complexity [54–56]. In addition, to enhance the therapeutic effects of bioengineered tissue constructs, there is a need to develop an intricate construction encapsulating various cell types that mimic the cellular diversity and complexity of injured tissue. With these increased demands, a traditional single biomaterial- and cell type-based scaffold may no longer meet various critical requirements for successful tissue regeneration [57]. Therefore, there is an urgent need to develop multifunctional modular tissue assemblies that can properly recapitulate the diversity of patient-specific tissue injury conditions, such as different cell types, structural complexity, size/shape, and multiple ECM components [58, 59]. In this context, the bottom-up assembly strategy of multifunctional tissue modules has recently attracted increased attention. In our Lego-like approach, the individually fabricated cell-embedded tissue blocks can be assembled into larger scalable tissue constructs with much higher cellular densities per unit area and can encapsulate more diverse cell types.

**In addition, the potential therapeutic mechanism of endometrial tissue assembly can be summarized as the following. Firstly, the customizable Lego-like tissue architecture developed in this study provides a complex patient-specific multicellular microenvironment that mimics the endometrial tissue. The presence of these different cell types is essential for endometrial tissue regeneration as they contribute to various functions such as angiogenesis, immune regulation, and hormone responsiveness. Secondly, the developed tissue assembly is made up of biodegradable biomaterials and certain endometrial constituent cells, including endometrial stem cells, myometrial cells, muscle cells, stromal cells, and vascular endothelial cells. These biomaterials and cells can interact with each other and contribute to the regeneration of damaged endometrial tissue. For example, biodegradable biomaterials such as collagen and hyaluronic acid can provide a structural framework and regulatory substances for increasing the survival and adhesion of the transplanted cells at the site of injury. Thirdly, the endometrial tissue secretes a diverse range of growth factors and cytokines (**Fig. 10A-B**), which significantly promote cellular functions such as proliferation, angiogenesis, and migration.**

Currently, various tissue modules can be produced by a number of procedures, including microfabrication techniques of cell-laden hydrogels [60], self-assembling aggregation [61], synthesis of cell sheets [62], or 3D bioprinting [63]. However, these strategies are not effective for clinically and commercially important mass production of various tissue modules due to quality inconsistency and batch-to-batch variations. **Indeed, our group previously developed a basic endometrial tissue mimetic by mixing natural polymer materials such as collagen and hyaluronic acid with several endometrial constituent cells and observed a notable therapeutic effect**in vivo [64]. **Although this endometrial tissue mimetic exhibited some therapeutic efficacy, it also had several functional and structural limitations. Firstly, the mimetic only comprised a simplified three-layer structure that was markedly distinct from the intricate physiological and structural characteristics of real endometrial tissue. Secondly, the tissue mimetic included only three types of endometrial cells (stem cells, vessel cells, and stromal cells), whereas the actual endometrium contains a multiple cell types including immune cells and myometrial cells. Thirdly, the distribution of embedded endometrial cells within the biomaterial-based matrix differed significantly from that of the actual endometrium. Rather than being concentrated in a specific area or following a certain pattern, the cells were evenly distributed throughout the tissue mimic. In this context, as an alternative, we developed a customizable modular tissue assembly that allows easy and scalable configuration of a complex patient-specific tissue microenvironment.** Furthermore, we integrated both traditional mold casting and 3D printing technologies in present study. Individual tissue blocks were synthesized by casting with 3D printed PLA mold platforms to effectively and stably produce a large amount of tissue blocks at a much faster time and at low cost than the previously reported tissue module producing approaches. Briefly, we injected a biomaterial mixture encapsulating certain types of endometrial cells into the casting molds for individual tissue blocks and then polymerized them at room temperature. Next, polymerized tissue blocks were effectively released from the casting mold by pushing them with a separator (Fig. 5A-F).

In contrast to traditional scaffold concepts, our multifunctional modular strategy resembles key features of the Lego brick concept, which allows easy and scalable configuration of complex 3D tissue architecture for increased versatility via intuitive manual block-building into higher-order biological constructs [65]. Indeed, multimodular endometrial tissue can be assembled not only by the same tissue block but also by different types of blocks. Customized endometrial tissue assembly reflecting patient-specific tissue conditions is created through a diverse combination of various tissue blocks, which encapsulate certain types of endometrial cells. Even though these Lego-like multimodular strategies are effective and recapitulate complex tissue architecture, several key challenges, such as assembly accuracy, joint stability, and sealing issues, still remain. Therefore, to meet these requirements, we assembled individually fabricated tissue blocks using an assembly sheet, which has exactly the same shape and size of empty slots as the individual tissue blocks. We formed a stably connected tissue assembly without leakage by inserting individual tissue blocks into the corresponding slot on the assembly sheet, similar to Lego bricks (Fig. 10A and B).

The fibrous structure of the extracellular matrix (ECM) plays a critical role in supporting cell viability, adhesion, matrix-mediated migration, self-renewal, and differentiation into different lineages [66, 67] and is thus a key component for biomaterial-based regenerative medicine as a tissue framework. Therefore, we employed major ECM components, such as collagen and hyaluronic acid, to make individual endometrial tissue blocks to provide not only structural and mechanical support for various encapsulated endometrial cellular components but also their biochemical interactions to enhance cell viability and adhesion. First, collagen-based porous tissue mimetics have been extensively studied for tissue regeneration due to their excellent properties, such as low immunogenicity, biodegradability, and biocompatibility [68]. Second, hyaluronic acid, a large nonsulfated glycosaminoglycan, is one of the key elements of the ECM and has an important role in tissue regeneration by regulating various cell behaviors, such as angiogenesis, cell growth, and inflammatory responses [69]. It also plays a pivotal role in the regulation of multiple cellular functions by suppressing cell/matrix–cell interactions or intracellular signaling pathways [70]. Although these natural polymers have high flexibility and biocompatibility, their mechanical strength is not sufficient to properly sustain various tissue-specific physical properties after implantation at the defect sites. Therefore, several chemical crosslinking agents, such as acyl azide, aldehydes, chromium reagent, or formaldehyde, have been applied to increase the mechanical strength and degradation properties of biomaterials [71, 72]. However, their critical disadvantage is the potential cytotoxicity of residual unreacted chemical crosslinking agents [21]. Therefore, to avoid the disadvantages associated with the use of chemical crosslinking agents, we adopted naturally occurring blood coagulation factors (fibrinogen and thrombin) as natural crosslinking agents to reinforce the mechanical strength of collagen and hyaluronic acid mixtures. Adding exogenous thrombin can mediate the enzymatical cleavage of fibrinogen to fibrin monomers, leading to the formation of more highly branched fibrin fibers [73], which in turn increases polymerization of the collagen and hyaluronic acid mixture. Indeed, we observed that exogenous addition of fibrinogen and thrombin reinforced mechanical strength, which was more similar to that of the native endometrial tissue (Fig. 3D). Moreover, these natural polymer-based tissue blocks with blood coagulation factors provided high cell viabilities (Fig. 7A), metabolic activities (Fig. 7B), and maintenance of various molecular characteristics (Fig. 8). Importantly, severe degenerative changes, including the loss of the epidermis and dermis layer, were remarkably relieved by the transplantation of the multimodular tissue assembly (Fig. 11C-E).

## Conclusions

In conclusion, we newly developed a customizable multimodular endometrial tissue architecture by assembling individually synthesized tissue blocks that encapsulate certain types of endometrial cells. Individual tissue blocks were synthesized by integrating both traditional mold casting and 3D printing technologies. Briefly, the biomaterial mixture (collagen and hyaluronic acid) and specific endometrial cell type were injected into the 3D printed casting mold and then polymerized. Next, individual tissue blocks were stably assembled into a multimodular endometrial tissue architecture by inserting tissue blocks into multiple empty slots on the assembly sheet. In this way, a customized endometrial tissue assembly is created through a diverse combination of different tissue blocks with a much faster time and at a lower cost than that of the previous module-making strategies. Remarkably, this customizable modular tissue assembly allows easy and scalable configuration of a complex patient-specific tissue microenvironment, thus accelerating various tissue regeneration procedures.

In addition, the customizable multimodular endometrial tissue assembly developed in this study has shown promising results in effectively regenerating damaged endometrial tissue in vivo. This approach has the potential to overcome the limitations of traditional scaffold-based tissue engineering approaches and stem cell-based therapies. Additionally, the use of natural crosslinking agents to reinforce the mechanical strength of the biomaterials used in this approach provides a safer alternative to traditional chemical crosslinking agents. The Lego-like modular design and mass producibility of the tissue blocks also make this approach a cost-effective and practical option for personalized tissue engineering. Further research and development of this approach could lead to improved clinical outcomes for patients with endometrial injury or infertility.

## Electronic supplementary material

Below is the link to the electronic supplementary material.

Supplementary Material 1

Supplementary Material 2

## Data Availability

Not applicable.

## Abbreviations

bFGF: Basic fibroblast growth factor
CAD: Computer aided design
CK14: Cytokeratin 14
ECM: Extracellular matrix
EGFR: Epidermal growth factor receptor
ELISA: Enzyme-linked immunosorbent assay
ER: Estrogen receptor
GEO: Gene expression omnibus
HGF: Hepatocyte growth factor
HUVECs: Human umbilical vein endothelial cells
IACUC: Institutional animal care and use committee
IPA: Ingenuity pathway analysis
IGFBP: Insulin-like growth factor-binding protein
PGE2: Prostaglandin E2
PLA: Polylactic acid
PR: Progesterone receptor
SEM: Scanning electron microscopy
VEGF-A: Vascular endothelial growth factor A
vWF: von Willebrand factor
