# Supplementary material for: Development of cell-laden multimodular Lego-like customizable endometrial tissue assembly for successful tissue regeneration
Source: Biomater Res. 2023 Apr 21;27:33. doi: 10.1186/s40824-023-00376-9 (PMC10122345; doi:10.1186/s40824-023-00376-9)
Supplement: Supplementary file 1 — Supplementary Material 1 [file 40824_2023_376_MOESM1_ESM.pdf]

Supplementary figure legends

Supplementary figure 1

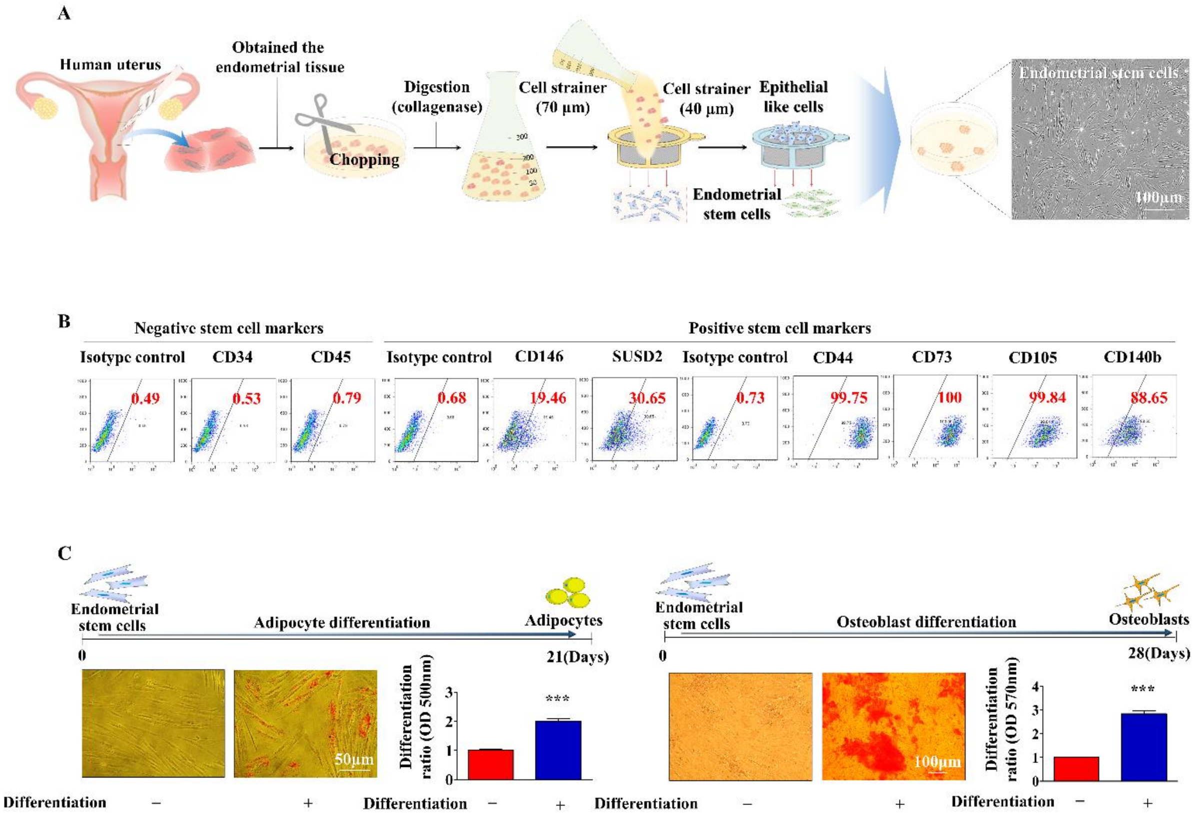

Supplementary Figure 1. Isolation and characteristic analysis of multipotent endometrial stem cells obtained from human endometrial biopsies. Human endometrial tissue specimens were minced into small pieces with a surgical blade and then enzymatically digested with type I collagenase solution for 6 hours. Isolated cells were observed using a phase contrast microscope to evaluate their growth and morphology **(A)**. The characteristics of isolated stem cells were evaluated using flow cytometry with many different antibodies for putative stem/progenitor cell markers (CD44, CD73, CD105, CD140b, CD146, and susD2) and two hematopoietic cell markers (CD34 and CD45) **(B)**. Their multilineage differentiation capacities into adipocytes and osteoblasts were assessed by performing oil red O staining for lipid contents and alizarin red S staining for calcium deposits in differentiated cells, respectively **(C)**. Significant differences are presented. *$p < 0.05$, **$p < 0.005$, and ***$p < 0.001$ (two-sample t-test).

Supplementary figure 2

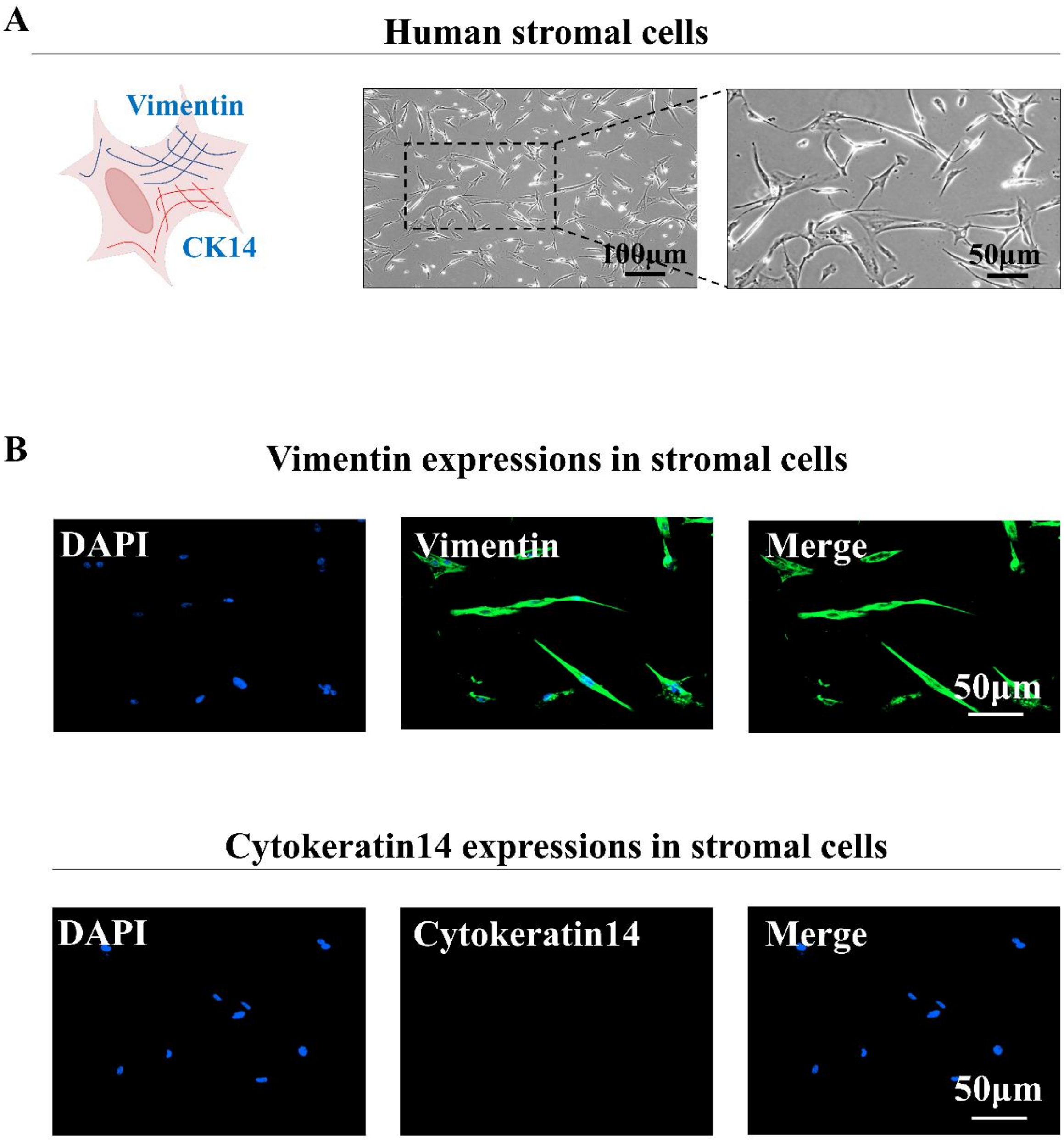

Supplementary Figure 2. Expansion and characteristic analysis of human stromal cells. Fibroblast-like human stromal cells isolated from normal skin biopsies were observed by phase contrast microscopy **(A)**. Their fundamental characteristics were analyzed by immunocytochemistry with several antibodies. These cells highly expressed the putative fibroblast marker vimentin and weakly expressed the well-known epithelial cell marker cytokeratin 14 (CK14) **(B)**. DAPI staining was used to label the nuclei within each field.

Supplementary figure 3

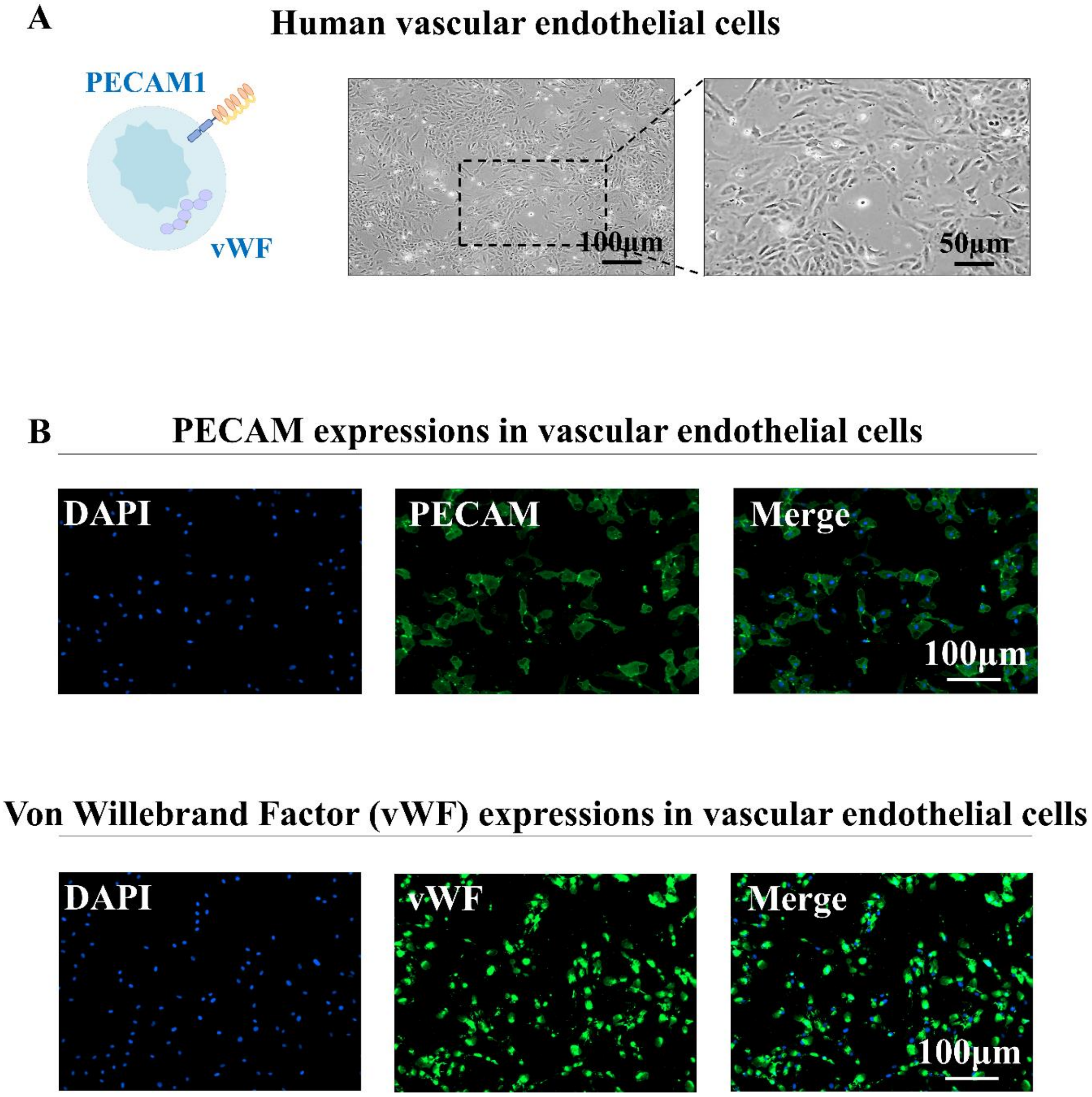

Supplementary Figure 3. Expansion and characteristic analysis of human vascular endothelial cells. Human umbilical vein endothelial cells (HUVECs) with polygonal shape characteristics were expanded and observed under phase contrast microscopy **(A)**. Their expression patterns of the specific endothelial cell markers PECAM-1 and vWF were analyzed by immunostaining with the corresponding antibodies **(B)**. DAPI staining was used to label the nuclei within each field.

Supplementary figure 4

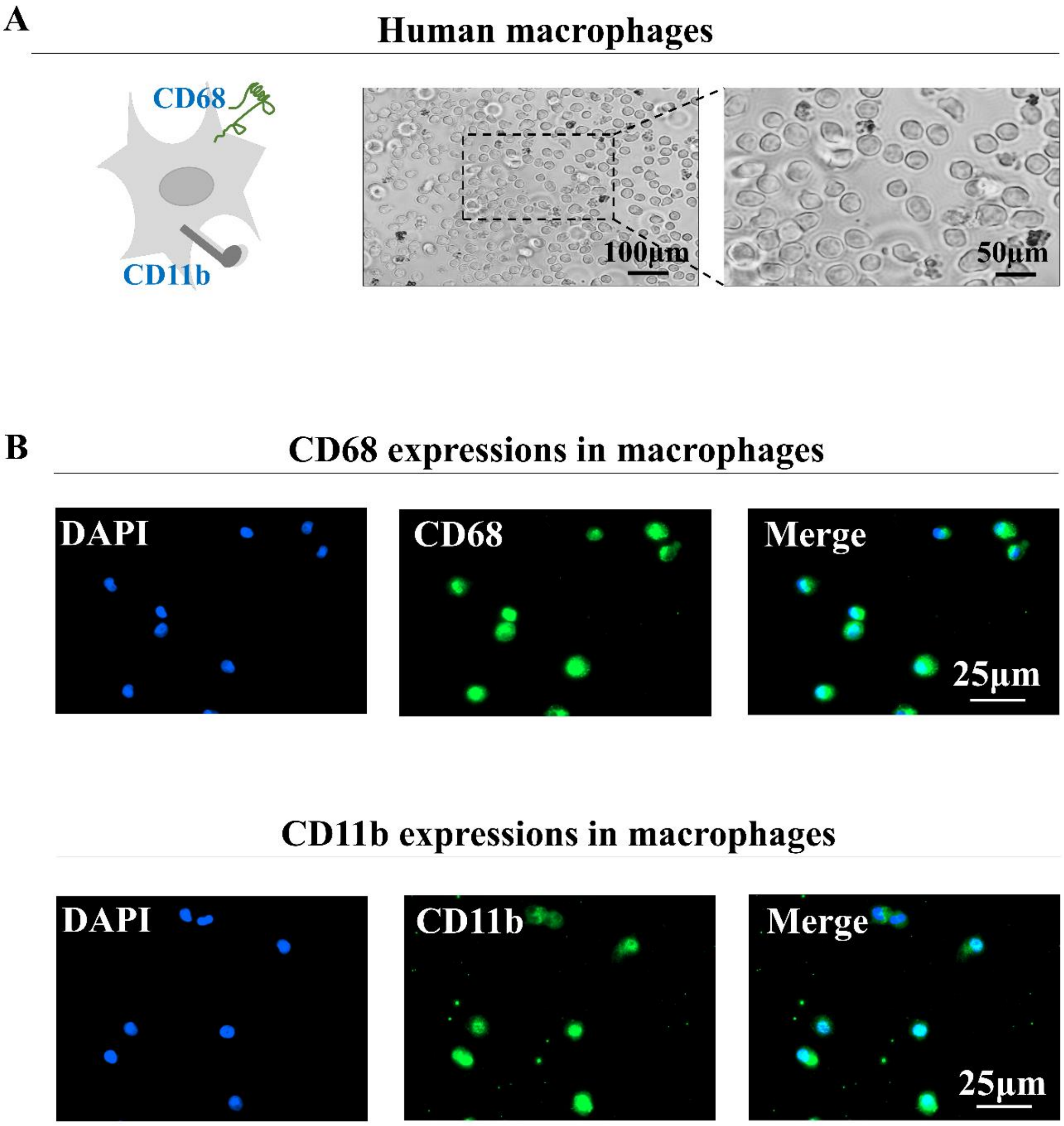

Supplementary Figure 4. Expansion and characteristic analysis of human macrophages. Macrophages with round shape characteristics were observed under phase contrast microscopy **(A)**. The expression patterns of the specific macrophage markers CD11b and CD68 were analyzed by immunostaining with the corresponding antibodies **(B)**. DAPI staining was used to label the nuclei within each field.

Supplementary figure 5

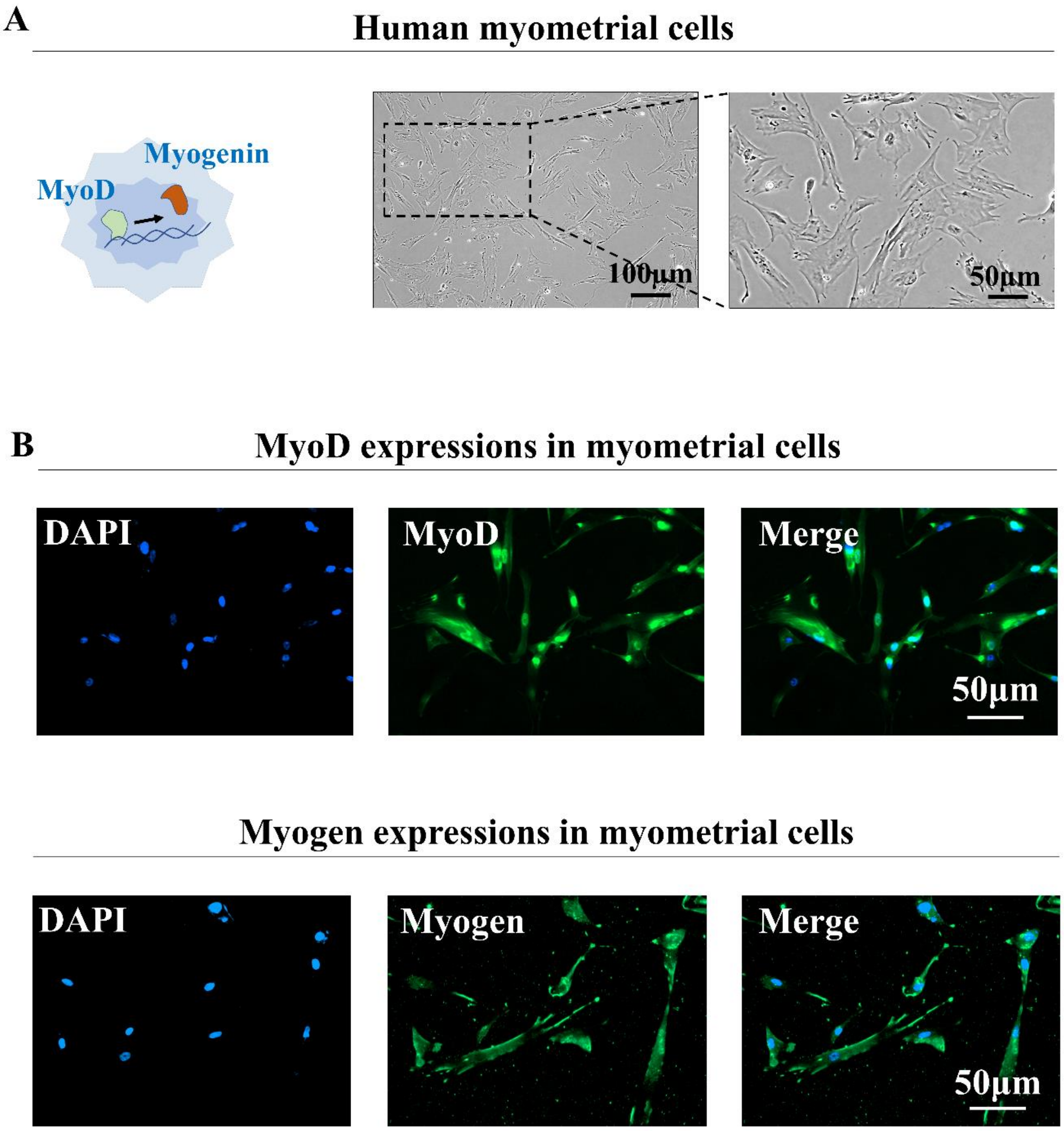

Supplementary Figure 5. Expansion and characteristic analysis of human myometrial cells. Human myometrial cells with spindle shape characteristics were observed under phase contrast microscopy **(A)**. The expression patterns of the specific myometrial cell markers Myo D and myogenin were analyzed by immunostaining with the corresponding antibodies **(B)**. DAPI staining was used to label the nuclei within each field.

Supplementary figure 6

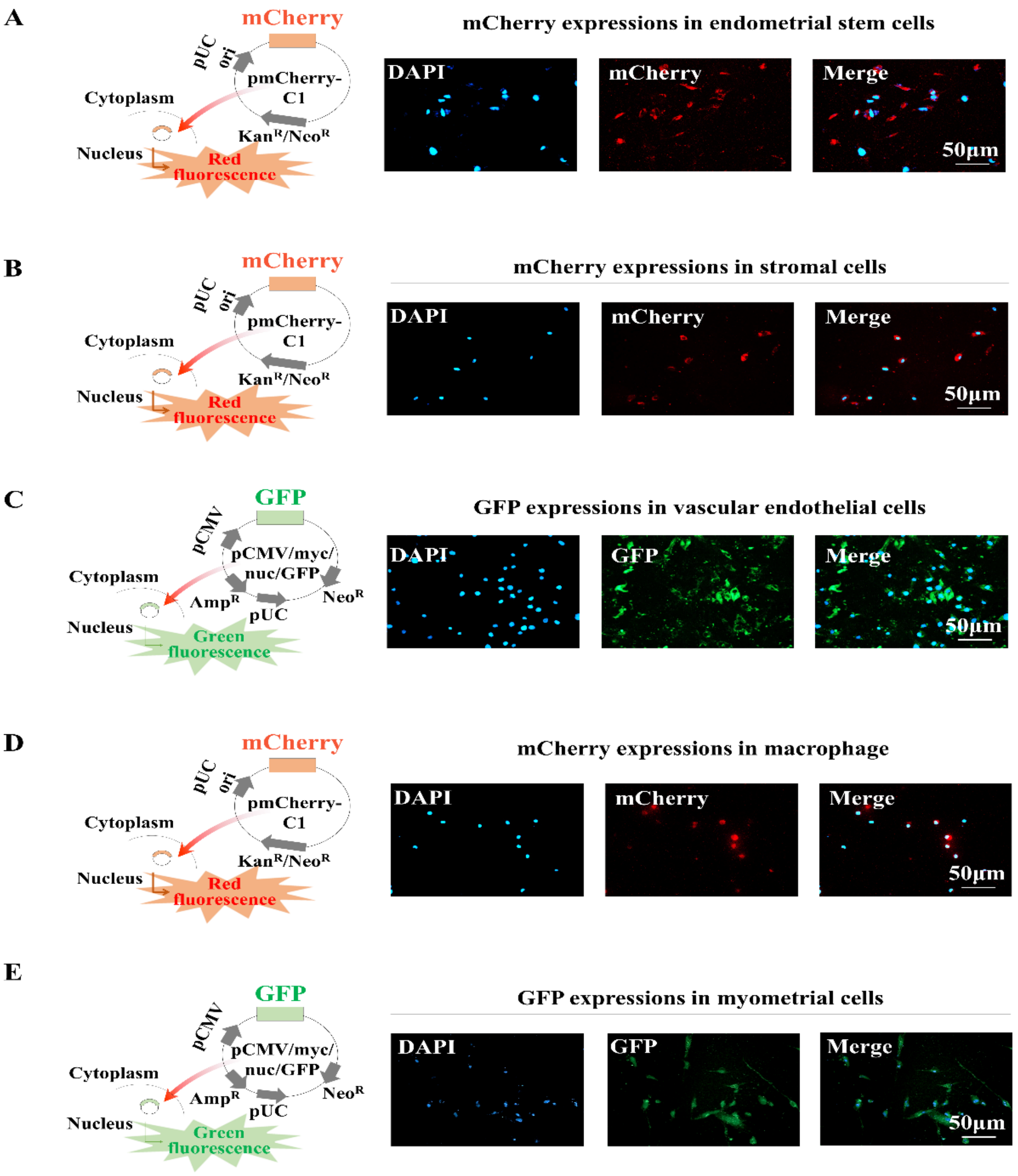

Supplementary Figure 6. Various cellular components of human endometrial tissue were stably labeled by transfection of a GFP- or mCherry-expressing vector. Cells embedded (1×10^6 cells/ml) within individual endometrial tissue blocks were transfected with fluorescent GFP- or mCherry-expressing vectors; endometrial stem cells were labeled with mCherry **(A)**, stromal cells were labeled with mCherry **(B)**, vascular endothelial cells were labeled with GFP **(C)**, macrophages were labeled with mCherry **(D)**, and myometrial cells were labeled with GFP **(E)**. The transfection efficiency was analyzed by immunostaining 48 h after transfection.

Supplementary figure 7

A

Positive correlation between vascular maturation and angiogenesis in endometrial tissue

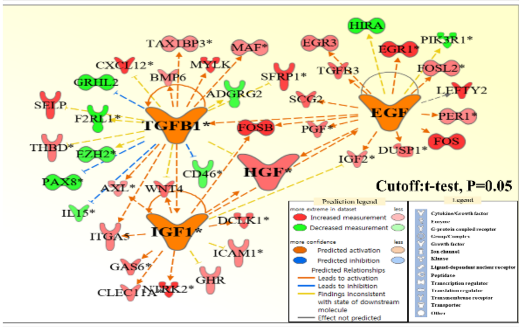

Signaling pathways	Predicted activation group	# of Target molecules in dataset	Predicted activation state	Activation z-score	P-value of overlap
**TGFB1**	Ectopic endometrial tissue (vs eutopic endometrial tissue)	206	Activated	5.085	7.63E-18
**EGF**	Ectopic endometrial tissue (vs eutopic endometrial tissue)	63	Activated	4.304	3.39E-06
**IGF1**	Ectopic endometrial tissue (vs eutopic endometrial tissue)	61	Activated	3.474	9.06E-05

B

Positive correlation between immune signals and response in uterus

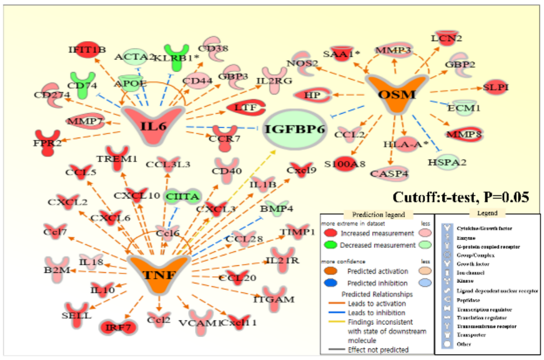

Signaling pathways	Predicted activation group	# of Target molecules in dataset	Predicted activation state	Activation z-score	P-value of overlap
**IL6**	Immune modulator treated in uterus (vs Non-treated in uterus)	26	Activated	2.070	3.01E-01
**OSM**	Immune modulator treated in uterus (vs Non-treated in uterus)	20	Activated	2.122	4.20E-02
**TNF**	Immune modulator treated in uterus (vs Non-treated in uterus)	62	Activated	2.344	3.32E-01

C

Negative correlation between vascular maturation and angiogenesis in uterine microvascular endothelial cells

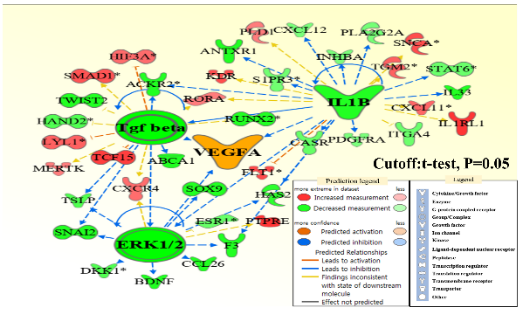

Signaling pathways	Predicted activation group	# of Target molecules in dataset	Predicted activation state	Activation z-score	P-value of overlap
**TGF beta**	Uterine microvascular endothelial cells (vs smooth muscle cells)	46	inhibited	-2.081	6.46E-13
**IL-1β**	Uterine microvascular endothelial cells (vs smooth muscle cells)	76	inhibited	-2.173	1.10E-12
**ERK1/2**	Uterine microvascular endothelial cells (vs smooth muscle cells)	31	inhibited	-2.096	2.13E-09

D

Negative correlation between vascular maturation and angiogenesis in uterine microvascular endothelial cells

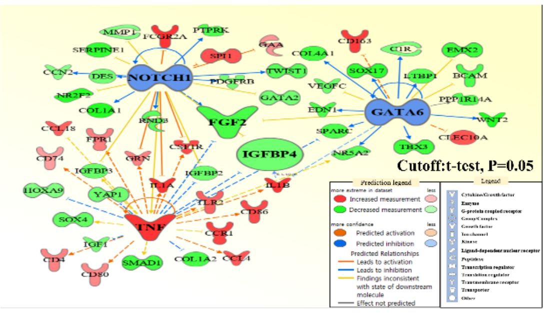

Signaling pathways	Predicted activation group	# of Target molecules in dataset	Predicted activation state	Activation z-score	P-value of overlap
**NOTCH1**	Uterine microvascular endothelial cells (vs smooth muscle cells)	51	inhibited	-2.788	2.91E-09
**GATA6**	Uterine microvascular endothelial cells (vs smooth muscle cells)	34	inhibited	-2.429	4.06E-08
**TNF**	Uterine microvascular endothelial cells (vs smooth muscle cells)	258	Activated	4.244	1.25E-31

Supplementary Figure 7. The signaling networks of secreted prominent factors from the multimodular endometrial tissue assembly are positively correlated with various endometrial functions. The differential activation status (either inhibited or activated) of multiple signaling networks, such as HGF (GSE25628), IGFBP6 (GSE85414), IGFBP4 (GSE3239), or VEGF (GSE3239)-associated genes, in various endometrial functions was analyzed using IPA software **(A-D)**.
