# Supplementary material for: Development of cell-laden multimodular Lego-like customizable endometrial tissue assembly for successful tissue regeneration
Source: Biomater Res. 2023 Apr 21;27:33. doi: 10.1186/s40824-023-00376-9 (PMC10122345; doi:10.1186/s40824-023-00376-9)
Supplement: Supplementary file 2 — Supplementary Material 2 [file 40824_2023_376_MOESM2_ESM.docx]

Dear Edition-in-Chief, Biomaterials Research

We greatly appreciate your thoughtful comments that improved our manuscript entitled “**Development of cell-laden multimodular Lego-like customizable endometrial tissue assembly for successful tissue regeneration**”. We have revised the manuscript as suggested by the reviewers. The corresponding changes are highlighted below in the same order as commented by the reviewers. Should you have any queries, please do not hesitate to contact us.

Sincerely yours,

**Reviewer #2:**

Almost comments were explained in detail, however, two questions were not cleared. Thus, these questions should be elaborated and this manuscript is suitable for publishing in the Biomaterials Research after minor revision.

1. Please add a section to discuss about the potential mechanism of tissue assembly for tissue regeneration.

☞ We appreciate the reviewer’s constructive comments regarding the potential therapeutic mechanism of multimodular tissue assembly. The potential therapeutic mechanism of endometrial tissue assembly can be summarized as the following. Firstly, the customizable Lego-like tissue architecture developed in this study provides a complex patient-specific multicellular microenvironment that mimics the endometrial tissue. The presence of these different cell types is essential for endometrial tissue regeneration as they contribute to various functions such as angiogenesis, immune regulation, and hormone responsiveness. Secondly, the developed tissue assembly is made up of biodegradable biomaterials and certain endometrial constituent cells, including endometrial stem cells, myometrial cells, muscle cells, stromal cells, and vascular endothelial cells. These biomaterials and cells can interact with each other and contribute to the regeneration of damaged endometrial tissue. For example, biodegradable biomaterials such as collagen and hyaluronic acid can provide a structural framework and regulatory substances for increasing the survival and adhesion of the transplanted cells at the site of injury. Thirdly, the endometrial tissue secretes a diverse range of growth factors and cytokines (Fig. 10A-B), which significantly promote cellular functions such as proliferation, angiogenesis, and migration. Therefore, based on your valuable remarks, we added following statement to the relevant section of ‘**Discussion**’ as follows: **“**In addition, the potential therapeutic mechanism of endometrial tissue assembly can be summarized as the following. Firstly, the customizable Lego-like tissue architecture developed in this study provides a complex patient-specific multicellular microenvironment that mimics the endometrial tissue. The presence of these different cell types is essential for endometrial tissue regeneration as they contribute to various functions such as angiogenesis, immune regulation, and hormone responsiveness. Secondly, the developed tissue assembly is made up of biodegradable biomaterials and certain endometrial constituent cells, including endometrial stem cells, myometrial cells, muscle cells, stromal cells, and vascular endothelial cells. These biomaterials and cells can interact with each other and contribute to the regeneration of damaged endometrial tissue. For example, biodegradable biomaterials such as collagen and hyaluronic acid can provide a structural framework and regulatory substances for increasing the survival and adhesion of the transplanted cells at the site of injury. Thirdly, the endometrial tissue secretes a diverse range of growth factors and cytokines (Fig. 10A-B), which significantly promote cellular functions such as proliferation, angiogenesis, and migration.**”**

2. The effect of Lego-like customizable endometrial tissue assembly was not compared with other methods to explain significant efficacy of tissue assembly for tissue regeneration.

☞ We appreciate the reviewer’s constructive comments regarding the comparison of	the effect of Lego-like customizable endometrial tissue assembly with other approaches. Our group previously developed a basic endometrial tissue mimetic by mixing natural polymer materials such as collagen and hyaluronic acid with several endometrial constituent cells (stem cells, vessel cells, and stromal cells) and observed a notable therapeutic effect *in vivo* (Park et al. Biofabrication. 2021 Aug 16;13(4). It is challenging to determine the difference in therapeutic efficacy between the presently developed tissue assembly and the previously developed tissue mimetics since we did not evaluate their therapeutic effects under the same experimental conditions. Although our developed endometrial tissue mimetic exhibited some therapeutic efficacy, it also had several functional and structural limitations. Firstly, the mimetic only comprised a simplified three-layer structure that was markedly distinct from the intricate physiological and structural characteristics of real endometrial tissue. Secondly, the tissue mimetic included only three types of endometrial cells (stem cells, vessel cells, and stromal cells), whereas the actual endometrium contains a multiple cell types including immune cells and myometrial cells. Thirdly, the distribution of embedded endometrial cells within the biomaterial-based matrix differed significantly from that of the actual endometrium. Rather than being concentrated in a specific area or following a certain pattern, the cells were evenly distributed throughout the tissue mimic. In this context, as an alternative, we developed a customizable modular tissue assembly that allows easy and scalable configuration of a complex patient-specific tissue microenvironment. Therefore, based on your valuable remarks, we added following statement to the relevant section of ‘**Discussion**’ as follows: **“**Indeed, our group previously developed a basic endometrial tissue mimetic by mixing natural polymer materials such as collagen and hyaluronic acid with several endometrial constituent cells and observed a notable therapeutic effect *in vivo* [64]. Although this endometrial tissue mimetic exhibited some therapeutic efficacy, it also had several functional and structural limitations. Firstly, the mimetic only comprised a simplified three-layer structure that was markedly distinct from the intricate physiological and structural characteristics of real endometrial tissue. Secondly, the tissue mimetic included only three types of endometrial cells (stem cells, vessel cells, and stromal cells), whereas the actual endometrium contains a multiple cell types including immune cells and myometrial cells. Thirdly, the distribution of embedded endometrial cells within the biomaterial-based matrix differed significantly from that of the actual endometrium. Rather than being concentrated in a specific area or following a certain pattern, the cells were evenly distributed throughout the tissue mimic. In this context, as an alternative, we developed a customizable modular tissue assembly that allows easy and scalable configuration of a complex patient-specific tissue microenvironment.**”**
